# Evidence for environmental filtering and limiting similarity depends on spatial scale and dissimilarity metrics

**DOI:** 10.1002/ecy.70244

**Published:** 2025-11-10

**Authors:** Maria A. Perez‐Navarro, Harry E. R. Shepherd, Joshua I. Brian, Adam T. Clark, Jane A. Catford

**Affiliations:** ^1^ Department of Geography King's College London London UK; ^2^ Department of Biology University of Graz Graz Styria Austria; ^3^ Fenner School of Environment & Society The Australian National University Canberra Australian Capital Territory Australia; ^4^ Present address: Center for Ecological Research and Applied Forestry (CREAF) Barcelona Spain

**Keywords:** community assembly, Darwin's naturalization conundrum, environmental filtering, exotic plant species invasions, functional traits, grassland secondary succession, invasion ecology, limiting similarity, phylogenetic distance, pre‐adaptation.

## Abstract

Darwin's theory of natural selection provides two seemingly contradictory hypotheses for explaining the success of biological invasions: (1) the pre‐adaptation hypothesis posits that introduced species that are closely related to native species will be more likely to succeed due to shared advantageous characteristics; (2) the limiting similarity hypothesis posits that invaders that are more similar to resident species will be less likely to succeed due to competitive exclusion. Previous studies assessing this conundrum show mixed results, possibly stemming from inconsistent study spatial scales and failure to integrate both functional and phylogenetic information. Here, we address these limitations using a 33‐year grassland successional survey at Cedar Creek Ecosystem Science Reserve (USA). We incorporate functional dissimilarities, phylogenetic distances, environmental covariates, and species origin data for 303 vascular plant taxa (256 native, 47 introduced), collected from 2700 plots. In contrast with other studies, we test both hypotheses at two fine spatial scales—neighborhood (0.5 m^2^) and site (~40 m^2^)—to better capture competition and environmental filtering, respectively. Findings related to Darwin's naturalization conundrum depended on spatial scale and dissimilarity metric. Our results agreed with the pre‐adaptation hypothesis at site scale (40 m^2^)—a much finer resolution than typically used to test the pre‐adaptation hypothesis—highlighting the role of environmental filtering. At the neighborhood scale (0.5 m^2^), support for the limiting similarity hypothesis emerged when using functional dissimilarity, while phylogenetic distance aligned with the pre‐adaptation hypothesis, demonstrating that different dissimilarity metrics can yield contrasting conclusions. In addition, native and introduced species showed different abundance patterns in relation to functional ranked dissimilarities, with introduced species reaching higher cover when they were taller than co‐occurring species, had higher leaf dry matter content (LDMC) and lower seed mass. Introduced species also reached high cover with higher soil N concentrations and a shorter time after colonization, relative to native species. Our results suggest that inconsistent findings related to Darwin's naturalization conundrum may arise from an overreliance on single dissimilarity metrics and the use of spatial scales failing to capture underlying ecological processes. This highlights the need for more nuanced methodologies when testing the pre‐adaptation and limiting similarity hypotheses.

## INTRODUCTION

Humans are introducing species beyond their native ranges at an ever‐increasing rate (Seebens et al., 2017) with a consequent rise in the impact of invasions on biodiversity, ecosystem function, human health, and economy (Mazza & Tricarico, 2018; Pejchar & Mooney, 2009; Pyšek, Hulme, et al., 2020). Despite considerable research into the characteristics and processes underlying the establishment and spread of introduced (exotic, non‐native, alien) species, much uncertainty about species invasion remains (Catford et al., 2019; Pyšek, Novoa, et al., 2020; Pyšek & Richardson, 2008). From the wide number of hypotheses attempting to explain species invasions (Catford et al., 2009), dissimilarity between introduced and native species—and associated hypotheses about pre‐adaptation and limiting similarity—is key (Catford et al., 2009; Enders et al., 2020; Lemoine et al., 2016).

The pre‐adaptation and limiting similarity hypotheses are deeply rooted in natural selection theory and are frequently known as Darwin's naturalization conundrum owing to their contrasting expectations regarding functional and phylogenetic similarity and invasion success (Cadotte et al., 2018; Darwin, 1859; Diez et al., 2008; Thuiller et al., 2010). The pre‐adaptation hypothesis is predicated on the importance of environmental filtering for community assembly. It predicts that introduced species more closely related to native species will be more successful than distantly related invaders because they are more likely to already possess traits that are suitable in the introduced range (Fridley & Sax, 2014; Lososová et al., 2015; Qian & Sandel, 2022). In contrast, the limiting similarity hypothesis supposes that biotic interactions, particularly competition, dominate community assembly. As such, more distantly related and functionally dissimilar introduced species will be more successful than closely related ones as they will be better able to avoid competition with natives and fill “empty niches” (Carboni et al., 2013; Catford et al., 2009; Fridley & Sax, 2014; Thuiller et al., 2010).

**FIGURE 1:**
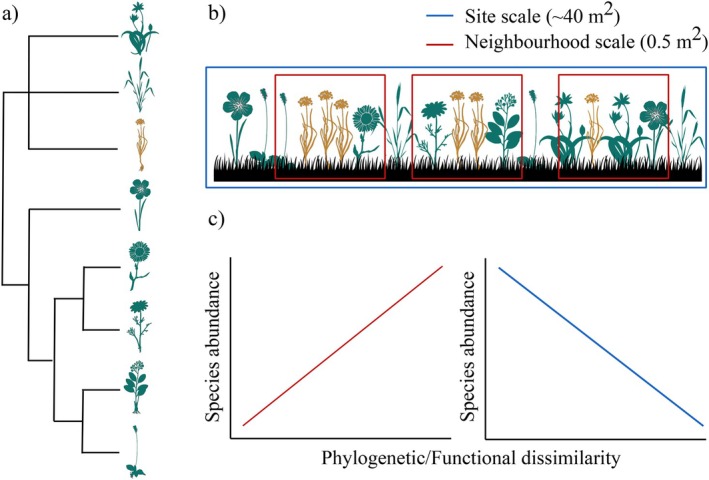
Summary diagram of expected support for pre‐adaptation and limiting similarity hypotheses at different spatial scales (i.e., spatial resolutions or grains). Each plant icon represents a different hypothetical herb species. Dark green color represents native species while ochre represents introduced species. Panel (a) shows the phylogenetic distribution of the species. Panel (b) represents the spatial distribution of the species in a given transect. Dark blue box represents the site scale (defined in this study as 40 m^2^). Red boxes represent the neighborhood scale (defined in this study as 0.5 m^2^). Panel (c) represents the expected relationship between species abundances and their phylogenetic/functional dissimilarity to the rest of the species present in the community. Dark blue trend at site scale would support the pre‐adaptation hypothesis (also known as environmental filtering) and red trend at neighborhood scale would support the limiting similarity hypothesis (also known as Darwin's naturalization hypothesis). Plant vector images were obtained from phylopic.org. under CC0 or Public Domain Mark 1.0 licenses.

Evidence for the pre‐adaptation and limiting similarity hypotheses may vary across spatial scales reflecting the differential influence of environmental filtering and competition on community assembly with scale (Cadotte et al., 2018; Gallien & Carboni, 2017; Götzenberger et al., 2012; Park et al., 2020). Generally, higher support for the pre‐adaptation hypothesis is expected at larger spatial scales, as increased environmental heterogeneity increases the strength of environmental filtering relative to biotic interactions (Figure 1) (Thuiller et al., 2010). In contrast, higher support for limiting similarity is expected at small spatial scales, as lower environmental heterogeneity at these scales increases our ability to detect plant–plant interactions (Figure 1) (Cadotte et al., 2018; Holden & Cahill, 2024; Kraft et al., 2015). However, these expectations are not always met, with different studies finding different trends (Cadotte et al., 2018), further complicating the conundrum. We suggest three reasons why support for the pre‐adaptation and limiting similarity hypotheses across spatial scales has been inconclusive.

First, some studies may use spatial scales that are inappropriate for the hypotheses and processes in question. For herbaceous plants, the spatial scale at which plant individuals are assumed to directly interact (i.e., “neighborhood scale” sensu Götzenberger et al., 2012) is thought to range from a few centimeters up to about 1 m^2^. Above this resolution, the relative importance of environmental filtering (i.e., pre‐adaptation) becomes more prominent, reducing the ability to detect effects of competition (i.e., limiting similarity) (Gotelli & Mccabe, 2002; Watkins et al., 2003). However, in numerous studies testing Darwin's naturalization conundrum, the smallest spatial scale ranges between 1 and 10 s of meters, while the larger scale typically ranges from a few km to continent‐wide (Cadotte et al., 2009, 2018; Lososová et al., 2015). This means that, for herbaceous plants, most studies are unlikely to detect effects of limiting similarity because the study scales exceed neighborhood scales (i.e., they are usually larger than 1 m^2^). A robust examination of Darwin's naturalization conundrum, that clearly tests both the pre‐adaptation and limiting similarity hypotheses, requires analysis of finer spatial scales.

Second, many studies may have failed to adequately represent species' dissimilarities by using *either* phylogenetic distance *or* trait dissimilarities as an indicator and by relying on absolute pairwise distance when functional dissimilarity is examined (Carboni et al., 2016; Catford et al., 2019). It is often assumed that phylogenetic distance captures the phenotypic expression of a whole plant (thus covering all traits at the same time) and that closely related species share similar traits (Cahill et al., 2008; Godoy et al., 2014). However, functional convergence and divergence can decouple the phylogeny–trait relationship, making phylogenetic distance an unreliable proxy of functional dissimilarity. Trait‐based dissimilarity measures can directly indicate functional differences, but this approach is usually limited to a few “soft” traits for which data are widely available (Belluau & Shipley, 2018). In addition, while absolute pairwise dissimilarities are thought to represent species' niches (Carboni et al., 2016, Catford et al., 2019), species' hierarchical position along a gradient of functional traits (ranked dissimilarities) better indicates competitive abilities (Gallien et al., 2015; Kunstler et al., 2012, 2016), constituting a more appropriate approach to test limiting similarity. Therefore, simultaneously examining phylogenetic and functional dissimilarity, and using ranked functional dissimilarity, would increase our capacity to capture species' ecological differences.

Third, important covariates may not have been considered or included in analyses, unintentionally masking effects of pre‐adaptation or limiting similarity and impeding effective hypothesis testing (Catford et al., 2022; Gallien & Carboni, 2017). For example, disturbance, such as fire or land use change, could weaken effects of limiting similarity by reducing plant competition (Catford et al., 2012; Kneitel & Perrault, 2006); unless disturbance history is explicitly considered in this example, support for the limiting similarity hypothesis might be misleadingly low.

Using a long‐term survey (1983–2016) of secondary successional grasslands in Minnesota, USA, here we overcome these common limitations by using an unusually comprehensive approach to examine Darwin's naturalization conundrum. Working at two fine spatial scales that correspond with competition and environmental filtering in grassland ecosystems, we combine (i) phylogenetic distance to represent species relatedness, (ii) ranked multivariate and univariate trait dissimilarities to represent species functional differences, and (iii) key environmental covariates to investigate support for the pre‐adaptation and limiting similarity hypotheses in both native and introduced species. Examining both native and introduced species can reveal whether all colonizing species are subject to the same processes regardless of species origin, or whether some trends are applicable to introduced species only (Lemoine et al., 2016). Specifically, we have two main expectations:

1. The limiting similarity hypothesis will gain most support at the neighborhood scale (0.5‐m^2^ plots) where individuals can directly interact and compete for resources, whereas the pre‐adaptation hypothesis will show higher support at site scale (~40‐m^2^ transects) where environmental filtering is likely to be stronger (Figure 1).
2. Introduced and native species will show similar responses to functional, phylogenetic, and environmental predictors, though introduced species might have different life history traits and higher competitive abilities than native species (Fridley & Sax, 2014; Gallien et al., 2015; Zheng et al., 2015).

More comprehensive tests of Darwin's naturalization conundrum will improve understanding of community assembly processes across spatial scales and provide useful insights for invasive species management.

## MATERIALS AND METHODS

### Study system

The study is based on data collected in a long‐term vegetation survey carried out at Cedar Creek Ecosystem Science Reserve, Minnesota (hereon Cedar Creek; 45.4° N, 93.2° W), within the experiment E‐014 (https://www.cedarcreek.umn.edu/research/data/methods?e014). The study site is part of a long‐term succession experiment and is characterized by nitrogen‐limited sandy soils, annual precipitation of 775 (±158 SD) mm, mainly concentrated between April and August, mean summer temperatures of 27°C (±1.6 SD), and winter lows of −14 (±1.2 SD)°C (according to closest weather station for the reference climate period 1963–2016), (see Inouye et al., 1987 for further details on site history).

Before widespread land clearing in the mid‐19th century, the study region was composed of a mix of grassland, oak savanna, deciduous forests, and wetlands (Cushing, 1963). Current vegetation in E‐014 is characteristic of secondary grasslands, with dominance of species from families *Asteraceae, Brassicaceae, Cyperaceae, Fabaceae, Poaceae, Polygonaceae*, and *Rosaceae*. Both native and introduced species are present, with introduced species contributing an average of 44% of plant cover across sample years.

### Field sampling and data preparation

Experiment E‐014 is divided into 26 fields, which were previously used as cropland for various species (including soybeans, rye, oats, and corn) and were abandoned in a staggered sequence between 1927 and 2015. The exact year of abandonment was obtained from historical documents and aerial photographs. Within each field, four permanent 39‐m‐long transects (hereafter site scale) were laid out, except for two fields where six transects were established. Each transect was divided into 25 plots of 0.5 m × 1 m (hereafter neighborhood scale), located every 1.5 m along the transect (with the first plot located at 1.5 m from the beginning of the transect) (Appendix S1: Figure S2). Heavy afforestation has occurred in all survey plots of one field and partially in plots of three other fields. Trees were absent or rarely appeared in the remaining 22 fields. When trees appeared, they were discarded from the corresponding plot and transect dataset, to keep only grassland communities. Finally, in all but four fields, half of the plots in each field were exposed to experimental periodic burning treatments since 2006, with a frequency of approximately one fire every 3 years (Clark et al., 2018).

Within the 0.5‐m^2^ plots, percent cover of every plant species, bare ground, and litter were visually estimated (see Clark et al., 2018 for further details). Eight vegetation surveys were conducted between 1983 and 2016, with surveys occurring roughly every 6 years mainly in summer (Clark et al., 2018). For each survey, the percent of cover of every plant species, bare ground and litter were visually estimated within the 0.5‐m^2^ plots. In total, 2700 plots (distributed within 108 transect, nested within 26 fields) were sampled, although some plots were not sampled in every survey. Across all years, a total of 16,944 plots surveys were conducted, encompassing 303 vascular plant species from which 218 were native, 47 were introduced and 38 had unknown origin (according to the species origin status recorded by the Agriculture Department of United States; USDA‐NRCS, 2022). This last category included both species with unknown origin status and species identified at genus level, the genera of which contain both introduced and native species in Minnesota. Overall, 77.9% of plots had at least one introduced species and in 46.1% of plots introduced species were dominant.

For each plot, soil nitrogen percentage, soil carbon percentage, soil organic matter concentration and light penetration were measured. Though these data were sampled in multiple years, we used average information across time, as soil and light sampled years do not match plant abundance surveys. In addition, we also estimated each species' minimum colonization time in each community. Minimum colonization time (hereafter colonization time) is similar to the concept of minimum residence time used in invasion ecology (Pyšek et al., 2004; Wilson et al., 2007) but refers to the minimum amount of time a species (whether native or introduced) was present in a given plot. We calculated colonization time as the difference between the year that a species was observed in a given plot and the year when the species first colonized the plot. Finally, succession time (i.e., time since plot abandonment; estimated as the difference between year of plot survey and year of field abandonment) and presence of burning treatment (Yes/No) were also considered in the analyses as proxies of disturbances (Kneitel & Perrault, 2006).

All described variables were estimated at both neighborhood and site scales. To obtain data at site scale, we averaged data for percent of plant cover; soil content in carbon, nitrogen and organic matter; and light penetration across all plots within the same transect and reestimated the minimum colonization time according with transect species composition and time records. Year of abandonment and burning treatment did not require scaling as these were already at the site scale. See Appendix S1: Section S1 for details of all methods.

### Trait data

Functional trait data for the study species were collected in grasslands and savannahs at Cedar Creek (Cadotte et al., 2009; Catford et al., 2019; Willis et al., 2010 and experiment E‐133). We selected seven functional traits: specific leaf area (SLA), plant height, leaf area, leaf dry matter content (LDMC), leaf fresh mass, leaf dry mass, and seed mass. Though these seven traits do not cover all the functional plant spectrum, they are assumed to cover most of the variability related to plant functional strategies (Díaz et al., 2016). We averaged trait data at the species level when more than one individual was sampled per species. We had plant height and leaf trait data for more than 60% of observed taxa, and seed mass data for 55% of observed taxa. We had complete data of all seven traits for 152 taxa. Although around 50% of the study taxa were not included in functional analyses (Appendix S1: Figures S3 and S4), trait representativeness at the plot level (neighborhood scale) and transect level (site scale) was high, with most of the plots and transects having trait data for more than 75% of the taxa, both in terms of the number of taxa and percentage cover (Appendix S1: Figure S5). See Appendix S1: Section S2 and Table S1 for details on trait data compilation and units, respectively. We also tested the phylogenetic signal of the seven traits for the study species (Appendix S1: Table S2), revealing a weak phylogenetic effect, thus ensuring a low correlation between functional and phylogenetic distances.

### Functional dissimilarity estimation

We applied both a multivariate functional approach, since it facilitates an integrated estimate of species functional traits (Martínez‐Vilalta et al., 2023) and a univariate approach to recognize the potential effects of specific traits. We followed two complementary approaches to estimate functional dissimilarity: (1) absolute pairwise distances to address the species' use of different niches and (2) ranked trait dissimilarities to address species' competitive hierarchical sorting (Carboni et al., 2016; Gallien & Carboni, 2017).

We first built a multivariate functional trait space by using principal components analysis (PCA, R package ade4, Dray & Dufour, 2007) to convert the functional space of the seven selected functional traits of all 152 species into a three‐dimensional volume defined by the first three PCA axes. Trait variables were scaled before conducting the PCA. The first three PCA axes explained 89.37% of the total variance (Appendix S1: Figure S6). Then, we obtained three PCA coordinates for each species. With these coordinates we estimated each species' dissimilarity to the rest of the species in its community by estimating (i) the three‐dimensional Euclidean distance to the community functional centroid (ranked multivariate dissimilarity) and (ii) pairwise absolute dissimilarity (pairwise multivariate dissimilarity). The community functional centroid was estimated as the center of gravity of the functional axis values of all species present in the community except the target species (i.e., the mean of species' coordinates in each PCA axis weighted by each species' cover percent; Appendix S1: Figure S7).

Similarly, we quantified ranked and pairwise functional dissimilarities for each species and each individual trait by calculating (i) the difference between each species' functional trait values and the community trait mean weighted by species cover (=ranked univariate functional dissimilarity) and (ii) the mean of absolute pairwise trait differences between target species and each species of the community (=pairwise univariate functional dissimilarity). Therefore, pairwise dissimilarities include no sign and indicate total trait distance between species regardless of trait sorting, and ranked dissimilarities include sign, where species with positive ranked functional dissimilarity indicate that the species have higher trait values than the average of the rest of the community while those having negative values have lower trait values than the rest of the community (Gallien & Carboni, 2017). Multivariate and univariate functional dissimilarities were estimated both at the neighborhood and site scale according to community composition in each case.

### Phylogenetic distance estimation

We used the V.phylomaker R package (Jin & Qian, 2019) based on Smith & Brown, 2018, and Zanne et al., 2014, phylogenies to obtain the phylogenetic tree of the total set of species present in the study area (Appendix S1: Figure S8). Species names were harmonized according to The Plant List using the taxonstand R package (Cayuela et al., 2021). From this phylogenetic tree with all the study species, we obtained phylogenetic trees for each plot and transect and estimated the phylogenetic distance between each species and the rest of the community as the weighted mean of each pairwise phylogenetic distance between the target species and each single species present in the community, weighted by the species' cover percent (Cadotte et al., 2010).

### Statistical analyses

We ran two sets of linear mixed‐effects models, one at plot level (neighborhood scale) and another at transect level (site scale) to assess the effect of phylogenetic and functional dissimilarity on species abundance. For both sets of models, we used percent cover as response variable. For matching normality criteria and dealing with 0 values, we transformed it as log(percent cover + 1). For explanatory variables, we used multivariate functional dissimilarity, univariate functional dissimilarity (seed mass, SLA, LDMC, leaf area, leaf fresh mass and dry mass, and plant height), phylogenetic distances, environmental conditions (soil nitrogen, soil carbon, soil organic matter content, burning treatment, and light penetration), temporal variables (minimum colonization time and succession time) and their interaction with species origin. Species origin was considered as a categorical variable with two categories: “introduced” (including only USDA introduced category) and “native” species (including both native species and species of unknown origin). There were only 16 unknown species from the total of 152 species included in the analyses. Univariate functional trait variables were scaled (subtracting the mean and dividing by SD), and the rest of the variables were logarithmically transformed when required to meet normality of model residuals (Appendix S1: Table S3). From this set of explanatory variables, we built alternative models (both at neighborhood and site scale), from a model including only phylogenetic and multivariate functional dissimilarity to a model including all the variables described above interacting with successional time (Clark et al., 2018) (Appendix S1: Table S3). Phylogenetic distance and functional dissimilarity variables were always included in the models since these variables were at the core of our analysis of Darwin's naturalization conundrum hypotheses. Collinearity between explanatory variables was tested for each model using Pearson correlation and variance inflation factor analyses (Ludecke et al., 2021), and highly correlated variables were removed from the fitted models. Species, year and plot nested within transect and transect nested within field were established as crossed random intercepts in case of neighborhood scale models; and species, year, and transect nested within field were established as crossed random intercepts for site scale models.

The final model was selected based on the corrected Akaike information criterion (AIC_c_) and Bayesian Information Criterion (BIC). It included all considered explanatory variables except soil carbon content and the interaction between successional time and rest of the variables (Appendix S1: Tables S4–S7). Final models were run both for ranked functional dissimilarities and pairwise functional dissimilarities separately. However, given the lower explanatory power of models including pairwise dissimilarity, only models including ranked functional dissimilarities are shown in the main text (but see Appendix S1: Figure S10 and Tables S6, S7, S10, S11, and S13). We also tested the final models using only plots and transects where species with trait data covered more than 90% of plant cover, in order to discard potential bias from missing trait data. We found no significant differences between these models and the ones that included all plots and transects, so we present the results based on the whole dataset. Linear mixed‐effects models were run using lme4 R package (Bates et al., 2015) and differences between species origin categories were assessed using emmeans R package (Lenth, 2021). All analyses and data manipulation were performed using R version 4.2.1 (R Core Team, 2022) following code from Perez‐Navarro, 2025.

## RESULTS

Relationships between species cover and our explanatory variables, including phylogenetic and functional dissimilarities, varied with spatial scale. However, the explanatory power of the fixed terms in the model (marginal *R* ^2^) was considerably lower than when random effects were also included (conditional *R* ^2^). Specifically, at neighborhood scale marginal *R* ^2^ was 7.7%, increasing to 35.8% for conditional *R* ^2^, and at site level marginal *R* ^2^ was 17.5%, increasing to 49.5% for conditional *R* ^2^ (Appendix S1: Tables S4 and S5).

### Support for limiting similarity and pre‐adaptation hypotheses across spatial scales according to multivariate functional dissimilarity and phylogenetic distance

**FIGURE 2:**
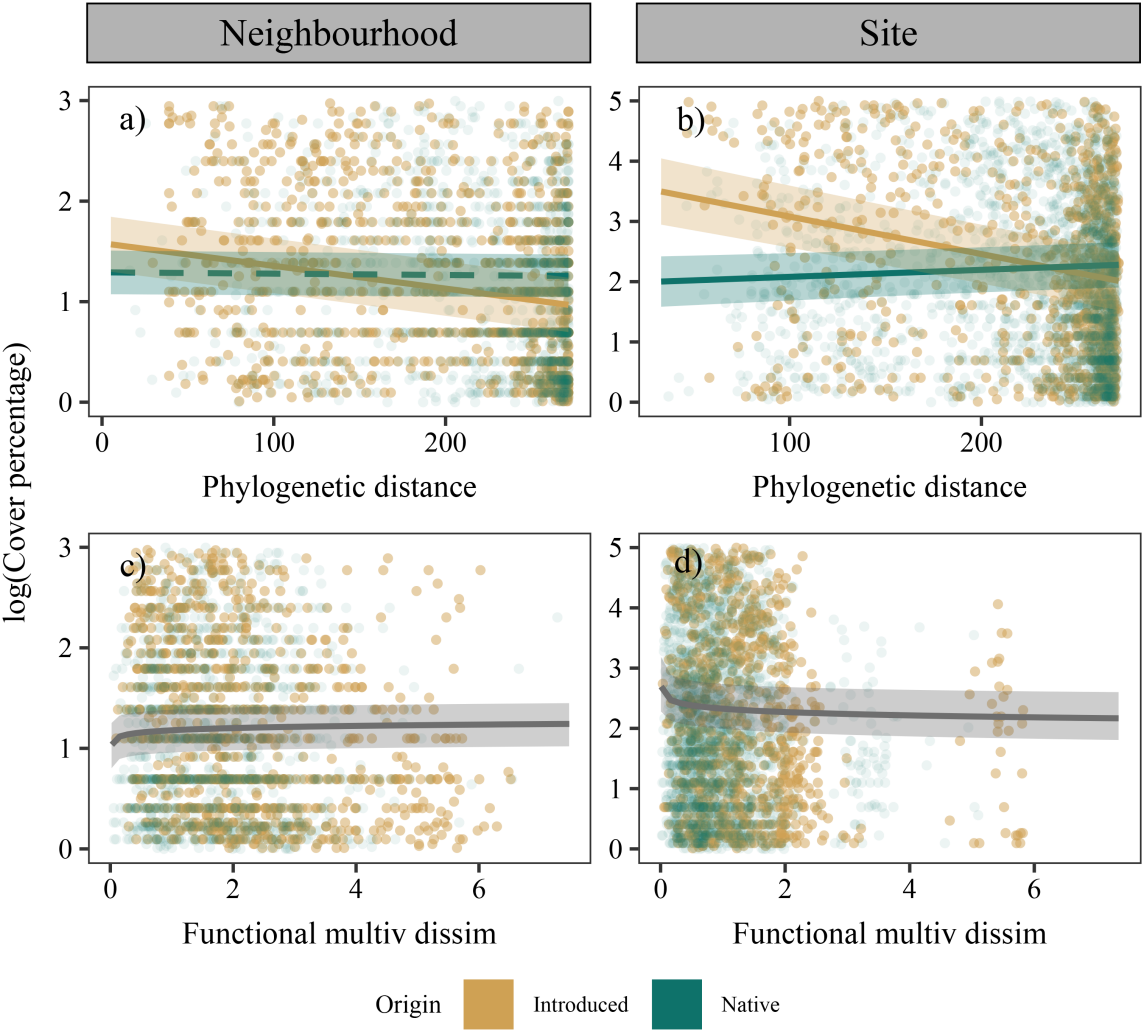
Regression plots from the final selected model for both neighborhood (0.5 m^2^) and site (~40 m^2^) spatial scales. *Y*‐axes represent log of species cover percent. *X*‐axes correspond to each of the variables used to evince Darwin's naturalization conundrum (phylogenetic distance and multivariate functional dissimilarity). Dark green color represents native species populations and ochre color represents introduced species populations. Gray color represents common trends for all the species when there were no significant differences between native and introduced species. Solid and dashed lines represent statistically significant (*p* < 0.05) and nonsignificant effects (*p* > 0.05), respectively. Plots only show 5% of species × plot raw data used in models at neighborhood scale and 25% of species × transect raw data at site scale, in order to reduce the number of points (to 5000 each) and improve visualization.

**FIGURE 3:**
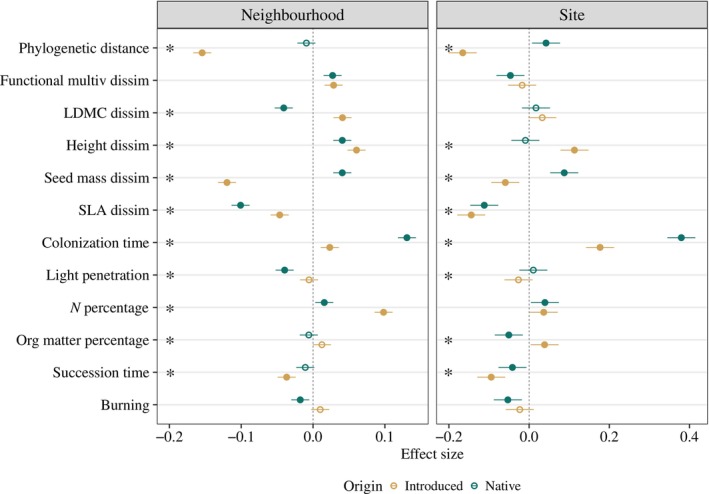
Effect size with 95% CIs of each single explanatory variable included in the final selected model both for neighborhood (0.5 m^2^) and site spatial scales (~40 m^2^). See Appendix S1: Table S3 for further details on model structure and variable transformation. Dark green dots represent native species, and ochre dots represent introduced species. Filled dots and empty dots represent statistically significant (*p* < 0.05) and nonsignificant effects (*p* > 0.05), respectively. Asterisk in the left of each panel indicates the presence of statistically significant differences between effect sizes of native and introduced species.

Relationships between species cover and multivariate functional dissimilarity varied with the spatial scale (Figure 2) and showed no significant interaction with species origin (Appendix S1: Tables S8 and S9). At the neighborhood scale, species that were functionally distinct from co‐occurring species in their communities reached higher cover than functionally similar species, similarly for both native and introduced species, consistent with the limiting similarity hypothesis (Figure 2c). In contrast, species more functionally similar to the rest of the community were more abundant at the site scale (Figure 2d), consistent with the pre‐adaptation hypothesis (though this trend was not significant for introduced species if we assess slope separately, Figure 3). In contrast, regarding phylogenetic distance, introduced species that were more closely related to co‐occurring species reached higher abundances than distantly related species at both spatial scales, providing support for the pre‐adaptation hypothesis (Figure 2a,b). Native species, however, showed a contrasting trend at the site level. Native species more distantly related to co‐occurring species showed higher cover than closely related natives (Figure 2b).

### Differences between introduced and native species abundance in response to univariate functional traits and environmental conditions

**FIGURE 4:**
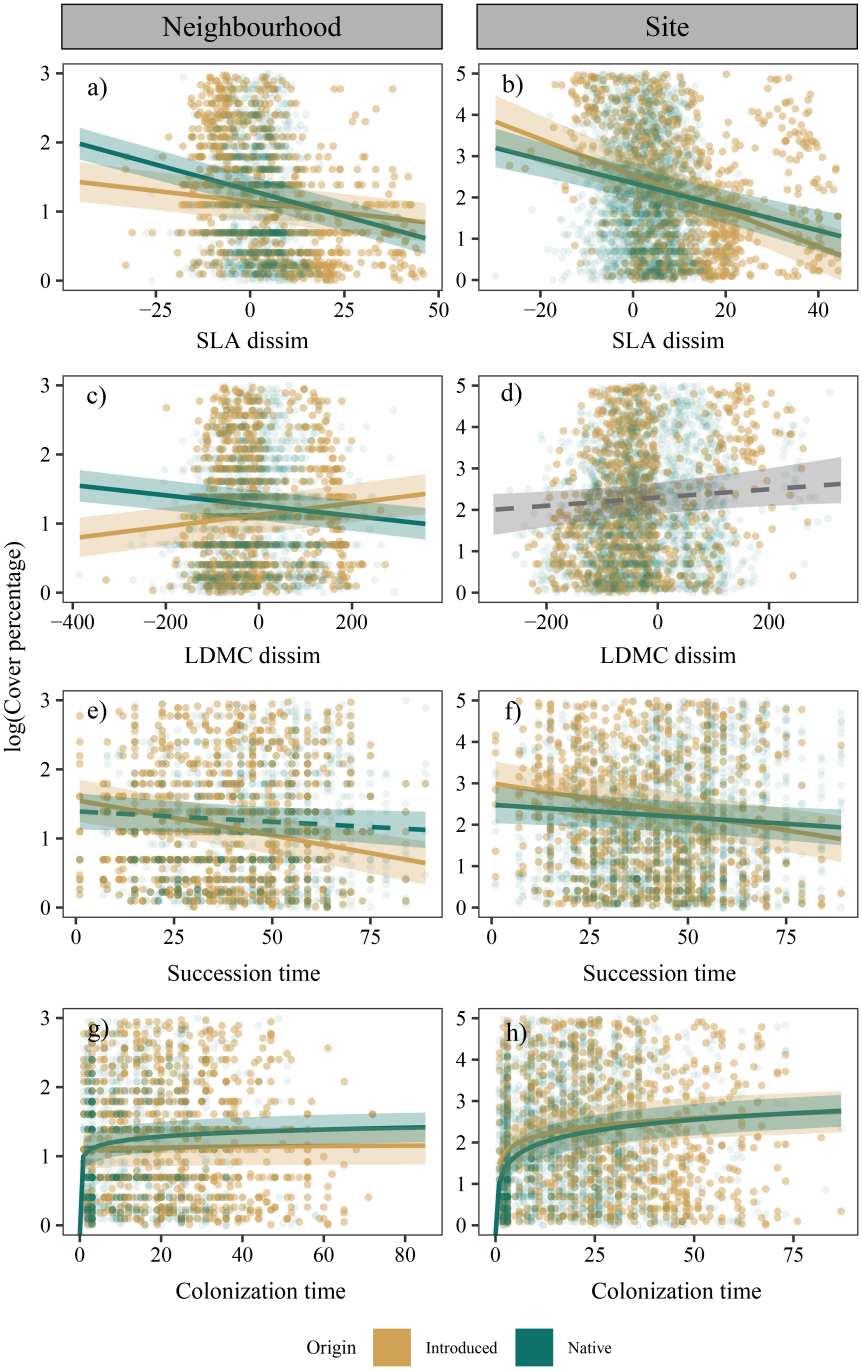
Regression plots from the final selected model for both neighborhood (5 m^2^) and site spatial scales (~40 m^2^). *Y*‐axes represent log of species cover percent. *X*‐axes correspond SLA and LDMC dissimilarities and succession time and species minimum colonization time. The rest of the traits and explanatory variables included in the final models can be seen in Appendix S1: Figure S9. Dark green color represents native species populations and ochre color represents introduced species populations. Gray color represents common trends for all the species when there were no significant differences between native and introduced species. Solid and dashed lines represent statistically significant (*p* < 0.05) and nonsignificant effects (*p* > 0.05) respectively. Plots only show 5% of species × plot raw data used in models at neighborhood scale and 25% of species × transect raw data at site scale, in order to reduce the number of points (to 5000 each) and improve visualization.

**FIGURE 5:**
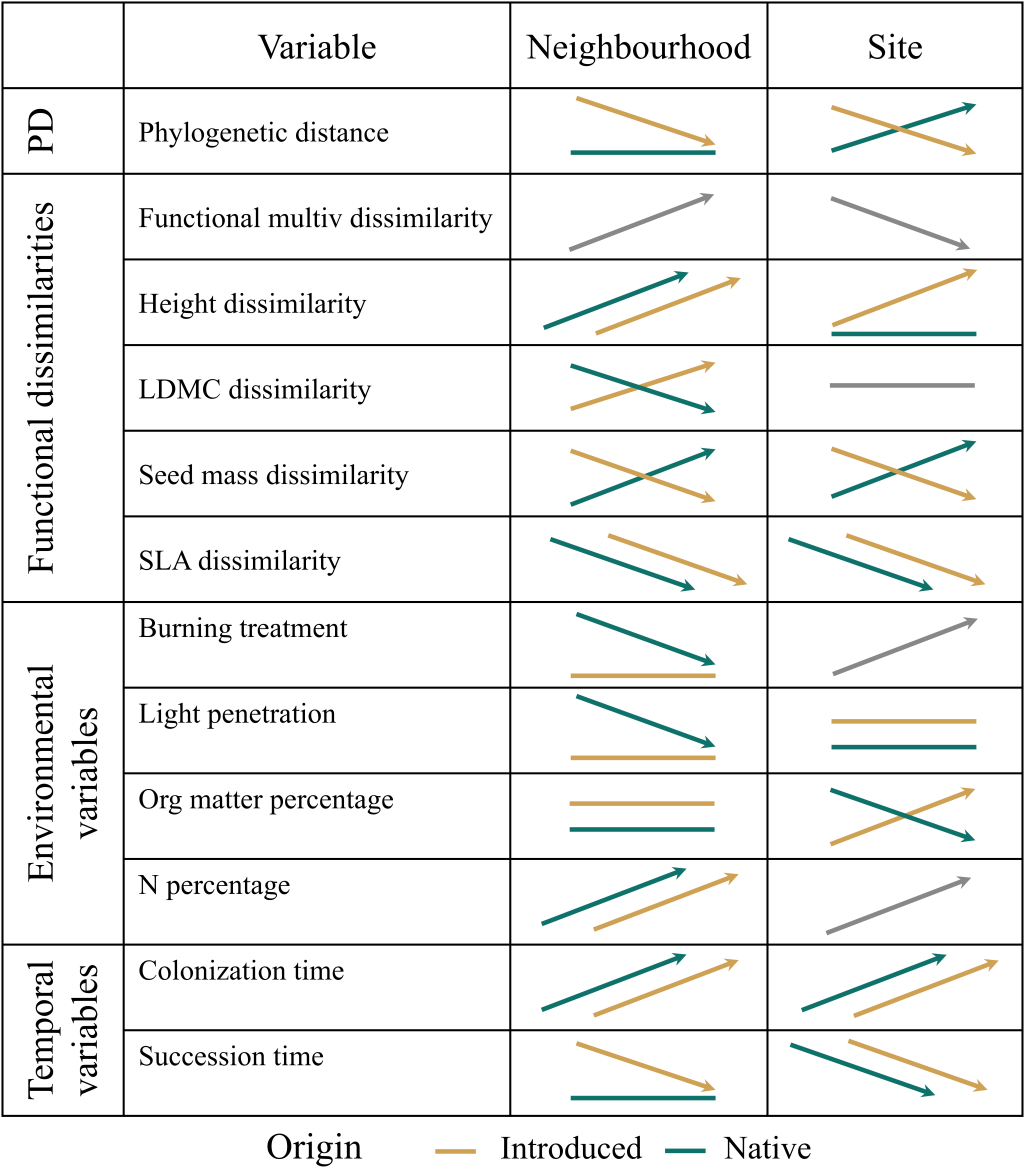
Visual summary of overall relationships between species abundances and the explanatory variables at neighborhood (5 m^2^) and site (~40 m^2^) scales. Figure is a visual aid for summary purposes only; actual relationships shown in Figures 2, 3, 4. For each panel, the *X*‐axis represents an increase in the listed variable, and the *Y*‐axis shows the response of species' percent cover. The slope of the arrows indicate the direction of observed trends (either positive or negative) but not its magnitude. Flat lines with no arrow heads indicate relationships that were not statistically significant. Dark green color represents native species and ochre color introduced species. Gray color represents common trends for all the species when there were no significant differences between native and introduced species.

Species origin showed a significant interaction with all ranked univariate trait dissimilarities and environmental variables, except for LDMC dissimilarity and soil nitrogen content at the site scale and burning treatment at both the neighborhood and site scale (Figure 4, Appendix S1: Figure S9 and Table S12). Some of the statistically significant differences between introduced and native species involved differences in relationship sign (direction), but most of these relationships only differed in strength (magnitude) (Figures 3, 4, 5, Appendix S1: Tables S8 and S9).

In the case of ranked trait dissimilarities, introduced and native species had contrasting relationships for LDMC at the neighborhood scale and seed mass at both scales (Figures 3, 4, 5). While native species showed higher cover with lower LDMC or heavier seeds, introduced species were more abundant when showing higher LDMC and lighter seeds than the rest of the species of the community (Figure 4c,d and Appendix S1: Figure S9c,d). For the other two ranked trait dissimilarities (height and SLA), introduced and native species' relationships differed in strength only (Figure 4a,b and Appendix S1: Figure S9a,b). Native and introduced species with higher size and lower SLA than the rest of the community tended to be more abundant (Figure 5).

Relationships between species cover and environmental variables were often not statistically significant, particularly at the neighborhood scale (Figures 3 and 5). When they were significant, introduced and native species' relationships usually differed in strength but not in sign (Figures 3, 4, 5 and Appendix S1: Tables S8 and S9). For example, the abundance of both native and introduced species increased with higher soil N concentrations at neighborhood scales, though introduced species abundance increased more (Appendix S1: Figure S9g, Table S8). Species abundances increased with colonization time and decreased with succession time both for native and introduced species, but introduced species had lower effect sizes for colonization time (Figure 4e–h). In contrast, introduced and native species had opposite relationships with soil organic matter concentration, with introduced species increasing and native species decreasing at the site scale (Figures 4 and 5 and Appendix S1: Figure S9j; Tables S9 and S12).

## DISCUSSION

Using a long‐term survey in secondary successional grasslands, we found that support for the two hypotheses underlying Darwin's naturalization conundrum varied depending on the study spatial scale and metric used to characterize species dissimilarities. Relationships between introduced species cover and their phylogenetic distance to the resident community were consistent with the pre‐adaptation hypothesis at both the neighborhood (0.5 m^2^) and site (40 m^2^) scales. In contrast, relationships between introduced species cover and multivariate functional dissimilarities aligned with the limiting similarity hypothesis at the neighborhood scale. In addition, in most cases, native and introduced species had distinct relationships with ranked univariate trait dissimilarities and environmental and temporal covariates, suggesting that, overall, introduced and native species possessed different life history strategies and competitive abilities.

### Support for limiting similarity and pre‐adaptation hypotheses across spatial scales

Consistent with expectations (Emery & Kellogg, 2007; Ma et al., 2016; Park et al., 2020), trends associated with multivariate functional dissimilarities mostly aligned with the limiting similarity hypothesis at the neighborhood scale (0.5 m^2^) and with the pre‐adaptation hypothesis at the site scale (~40 m^2^). These trends were similar for both native and introduced species, though the latter was weaker for introduced species at the site scale (Figure 3). The fact that the relationship between species abundance and functional dissimilarity differed across spatial scales as small as 0.5 and 40 m^2^ suggests that spatial scales of tens of meters—frequently used to represent local conditions in other studies (Götzenberger et al., 2012)—might be too large to evidence limiting similarity. Variation in spatial scales across studies likely contributes to the mixed conclusions about Darwin's naturalization conundrum in the literature (Cadotte et al., 2018; Catford et al., 2022, Hung et al., 2025).

Relationships between introduced species abundance and phylogenetic distance provided support for the pre‐adaptation hypothesis at both spatial scales, such that introduced species closely related to co‐occurring species had higher abundances (Figures 2 and 3). In contrast, native species showed no statistically significant trend at the neighborhood scale and showed higher abundances with larger phylogenetic distances at the site scale. Multiple studies that have examined Darwin's naturalization conundrum using phylogenetic distances have obtained higher support for the pre‐adaptation hypothesis at larger scales compared with finer scales or shown inconclusive results (Cadotte et al., 2018; Carboni et al., 2013; Ma et al., 2016; Park et al., 2020). Given the relatively fine resolution of spatial scales analyzed in our study, our results disagree with some of these previous studies by supporting the pre‐adaptation hypothesis at fine spatial scales when considering phylogenetic distances to characterize dissimilarities.

### Differences between functional and phylogenetic dissimilarities

Somewhat unexpectedly, we found simultaneous support for the pre‐adaptation and limiting similarity hypothesis at the neighborhood scale when using phylogenetic distance and functional dissimilarity, respectively. Though it is frequently assumed that closely related species should exhibit greater functional dissimilarity (Cahill et al., 2008), our results showed a weak correlation between phylogenetic and functional measures. This pattern might emerge due to (1) the use of traits with weak phylogenetic signal (Appendix S1: Table S2) (Flynn et al., 2011) because of evolutionary trait divergence of closely related species or convergence among distantly related taxa (Carboni et al., 2014) and (2) species colonization occurring in a stepwise process where the introduced species more closely related to natives are able to survive in the environmental conditions of the new range and then those more functionally similar species from these taxa are removed at finer scales due to competitive exclusion (Divíšek et al., 2018). Given the distinct trends of both metrics, our findings highlight the importance of examining both functional and phylogenetic dissimilarities when assessing Darwin's naturalization conundrum as one is not necessarily a reliable proxy of the other (Cadotte et al., 2013; Gallien & Carboni, 2017; Galland et al., 2019; Pinto‐Ledezma et al., 2020).

### Differences between native and introduced species

Our study shows that introduced and native species abundance can have similar relationships with some drivers, but distinct or opposite relationships with others. Introduced and native species had similar trends for multivariate functional dissimilarities, suggesting that limiting similarity influences community assembly regardless of species origin. This result aligns with assertions that native and introduced species have to follow the same set of “rules” to become abundant (Lemoine et al., 2015, 2016). However, phylogenetic distance, most ranked univariate functional dissimilarities and environmental and temporal variables revealed different trends depending on species origin. For example, introduced species with lighter seeds and lower LDMC were more abundant at neighborhood scales while native species showed the opposite trend. This suggests that introduced species benefitted from small seeds that can spread large distances (Richardson & Pyšek, 2006) and enhanced resistance to leaf desiccation (Pinto‐Ledezma et al., 2020). In addition, introduced species reached higher abundance in plots with higher soil nitrogen and organic matter content (Figure 3 and Appendix S1: Figure S10), consistent with ideas related to resource availability (Catford et al., 2009). The abundance of introduced species also increased more quickly than native species following land abandonment, thus implying that introduced species are better colonizers (Catford et al., 2012, 2018). These contrasted trends suggest that, depending on biogeographic origin, species can show different responses to ecological forces and possess distinct life history strategies (Zheng et al., 2015), highlighting the value of considering species' origin when assessing performance and ecological impacts (Buckley & Catford, 2016).

### Study limitations

The explanatory capacity of our models' fixed effects was considerably low, particularly at the neighborhood scale, despite our exhaustive approach including field data at fine spatial resolutions and a high number of hypothesis‐driven explanatory variables. A relevant amount of variability (28.1% at the neighborhood scale and 32% at the site scale) was captured by random effects in our models. This suggests that other variables related to species, plot and site identity, and interannual variability could help explain plant cover in our study. These might include, for example, functional dissimilarity for traits not considered in our analyses (e.g., root traits, Galán Díaz et al., 2023), species R* (Tilman, 1985) or small shifts in environmental conditions affecting competitive outcomes (Van Dyke et al., 2022; Catford et al., 2023). Other non‐deterministic variables, such as random propagule dispersion and demographic stochasticity (Hubbell, 2001; Tilman, 2004), could affect species abundance and account for the remaining unexplained variance, particularly at the neighborhood scale (Clark et al., 2018).

## CONCLUSION

By examining hypotheses underpinning Darwin's naturalization conundrum across two spatial scales in a long‐term field experiment, we found evidence for the limiting similarity hypothesis at the neighborhood scale (0.5 m^2^) and the pre‐adaptation hypothesis at the site scale (~40 m^2^), particularly when considering functional dissimilarity. This suggests that environmental heterogeneity strongly influences grassland community assembly even at very small spatial scales in grasslands. Importantly, this implies that spatial scales of a few tens of meters, frequently used as the “local” scale in studies examining the conundrum, can often be too large to exclusively show competition processes. Thus, many of the inconsistencies around support for the limiting similarity hypothesis in the literature could arguably stem from inappropriate selection of study spatial scales. Our results also revealed how support for hypotheses can depend on the variables used to examine them. Multivariate functional dissimilarity and phylogenetic distance each supported different hypotheses at the neighborhood scale, even though these two variables are often considered to be proxies for one another. Thus, we highlight that (i) the inappropriate selection and comparison of study spatial scales that fail to capture the corresponding community assembly process (competition or environmental filtering) and (ii) the independent use of either functional or phylogenetic approaches might cause the apparent context dependence and lack of consensus surrounding Darwin's naturalization conundrum. We also found that introduced and native species frequently had different relationships with environmental covariates and differed in terms of ranked trait position driving high abundance, suggesting that species' responses to environmental conditions and functional strategies can vary depending on species origin. Therefore, our study highlights the relevance of using appropriate spatial scales and multiple dissimilarity metrics when examining the complex interaction between species' biogeographic origin, functional strategies, and evolutionary history.

## AUTHOR CONTRIBUTIONS

Maria A. Perez‐Navarro conceptualized the original idea, led the data curation, did the formal analyses, and wrote the first draft. Adam Clark collected data, advised on data curation and analytical models. Josh Brian and Harry Shepherd advised on figure representation and analytical models. Jane Catford  conceptualized the idea, collected data, acquired funding and advised on figure representation and analytical models. All authors helped write later versions of the manuscript.

## CONFLICT OF INTEREST STATEMENT

The authors declare no conflicts of interest.

## Supporting information


Appendix S1.

## Data Availability

Plant cover and environmental data for experiment E‐014 of Cedar Creek Ecosystem Science Reserve are available in the following Environmental Data Initiative (EDI) releases: plant species percent cover data (Knops, 2024a), https://doi.org/10.6073/pasta/f0a2a36d882d9c937f817b15553e69ee; percent light penetration (Knops, 2018a), https://doi.org/10.6073/pasta/d145de38c27723e3e51c588b1dae751e; soil carbon (Knops, 2024b), https://doi.org/10.6073/pasta/acfd3e47f7cd4dff2f6fcd260fe9e623; soil nitrogen (Knops, 2024c), https://doi.org/10.6073/pasta/def568ca3242cfc504f534e2d55ecf67; soil organic matter (Knops, 2018b), https://doi.org/10.6073/pasta/b88c793cc4fdc0eeb232a46b3ddd169d. Phylogenetic information is included in the V.PhyloMaker package (Jin & Qian, 2019). Trait data have been obtained from previous studies (Cadotte et al., 2009; Catford et al., 2019; Willis et al., 2010). Data from Catford et al., 2019 are available through the TRY Plant Trait Database (https://www.try-db.org/TryWeb/Home.php) under dataset ID 354. Data provided by Cadotte et al. (2009) and Willis et al. (2010) are not the property of the authors of this paper to release publicly; these trait datasets should be requested from the corresponding author of those studies, and processed final data are available from the authors of this work. Additional trait data from experiment E‐133 of the Cedar Creek LTER are available in EDI in Reich (2018) at https://doi.org/10.6073/pasta/5814d764417a40dd80e56434b13a10b9. Code (Perez‐Navarro, 2025) is available in Zenodo at https://doi.org/10.5281/zenodo.15624601.
