# Supplementary material for: Evidence for environmental filtering and limiting similarity depends on spatial scale and dissimilarity metrics
Source: Ecology. 2025 Nov 10;106(11):e70244. doi: 10.1002/ecy.70244 (PMC12602181; doi:10.1002/ecy.70244)
Supplement: Supplementary file 1 — Appendix S1. [file ECY-106-e70244-s001.pdf]

Appendix S1

Evidence for environmental filtering and limiting similarity depend on spatial scale and dissimilarity metrics

Authors: Maria A. Perez-Navarro; Harry E. R. Shepherd; Joshua I. Brian; Adam T. Clark; Jane A. Catford

Ecology

Supporting Information

Section S1. Further details on field sampling and data preparation 4

Estimation of soil N, C, organic matter percentage and light penetration 4

Estimation of Minimum Colonisation Time 4

Section S1-Figure S1. Density plot of time lapse between cropland abandon and first documented occurrence in a given plot for different species 6

Section S2. Further details on trait data 7

Section S3. Appendix References 7

Figure S2. Transect spatial structure 9

Figure S3. Percentage species with data for each trait variable per origin status 10

Figure S4. Number of species with trait data regarding to source trait dataset 11

Figure S5. Proportion of species with functional trait data at plot and transect level from the total number of species present within each plot or transect 12

Figure S6. Correlation circles obtained from PCA for the 7 selected functional traits 13

Figure S7. Diagram representing the approaches used to measure the functional dissimilarity in the three-dimensional (3D) functional multivariate trait space 14

Figure S8. Phylogenetic tree for all the 303 species included in e014 Cedar Creek experiment database based on Smith and Brown 2018 and Zanne et al. (2014) phylogeny 15

Figure S9. Regression plots corresponding to final selected model for both neighbourhood and site spatial scales estimating dissimilarity as ranked trait differences. 17

Figure S10. Effect size with 95% confidence intervals of each single explanatory variable included in the final selected model both for neighbourhood (0.5 m2) and site spatial scales (~40 m2) considering dissimilarity as pairwise absolute differences 19

Table S1. Definitions and units of measurement of traits included in the study. 20

Table S2 Results of Blomberg's K test to assess the phylogenetic signal of the seven functional traits used to build the multivariate functional space. The table also includes the Pearson's correlation between multivariate functional dissimilarity (FMD) and phylogenetic distance (PD). 21

Table S3. Variables included in each of the 6 tested alternative models. 22

Table S4. Model summary for each of the fitted models at neighbourhood level (plot scale) using ranked functional univariate dissimilarities. 23

Table S5. Model summary for each of the fitted models at site level (transect scale) using ranked functional univariate dissimilarities. 24

Table S6. Model summary for each of the fitted models at plot level (neighbourhood scale) using absolute pairwise functional univariate dissimilarities. 25

Table S7. Model summary for each of the fitted models at site level (transect scale) using absolute pairwise functional univariate dissimilarities. 26

Table S8. Model results for the finally selected model at neighbourhood spatial scale including ranked functional dissimilarities. 27

Table S9. Model results for the finally selected model at site spatial scale including ranked functional dissimilarities. 28

Table S10. Model results for the finally selected model at neighbourhood spatial scale including absolute pairwise functional dissimilarities. 29

Table S11. Model results for the finally selected model at site spatial scale including absolute pairwise functional dissimilarities. 30

Table S12. Differences in slope between introduced and native species slopes for each variable (introduced slope-native slope) estimating functional dissimilarities as ranked traits dissimilarities. . 31

Table S13. Differences in slope between introduced and native species slopes for each variable (introduced slope-native slope) estimating functional dissimilarities as absolute pairwise dissimilarities. 32

Section S1. Further details on field sampling and data preparation

Estimation of soil N, C, organic matter percentage and light penetration

Soil organic matter, nitrogen and carbon content were measured at plot level at the beginning of the surveys in 1983, and soil nitrogen and carbon were measured every 6 years until 2011. Both nitrogen, carbon and organic matter concentrations were obtained as dry mass percentage, from one soil core of 25 mm x 100 mm located at the centre of each plot. Chemical analyses were conducted using a Carlo Urba NA 1500 elemental analyser (Knops & Tilman 2000; Knops & Bradley 2009). Light penetration was recorded in 1984, 1989 and 2016 at transect and plot level, respectively. In each case a pair of light meter readings were taken, one at ground level and one above the vegetation. Then, the light penetration percentage was obtained as light at ground level/light above vegetation*100. Since some plots and transects lacked light and soil data, we interpolated plot and transect level data when required to fill the gaps. When soil and light data were missing at plot level, average data from the rest of plots of the same transect were used. When transect data were missing, average data from the rest of transects within the same field were used. Since soil and light sampled years do not match the exact years of plant abundance surveys, we have averaged temporal soil and light data at plot or transect level.

Estimation of Minimum Colonisation Time

Minimum colonisation time in the community was estimated as the as the difference between the year that a species was observed in a given plot and the year when the species first colonised the plot. The year of first colonisation was estimated as the midpoint year between the year the species was observed the first time in the plot and the previously sampled year in the plot, when the species was still not present. For example, if a given species appeared in a plot for the first time in 1989 and the plot was previously sampled in 1983, we would infer 1986 as colonisation year. We used this approach instead of using the first-observed record as colonisation year (Castro et al., 2005; Wilson et al., 2007) since not all plots were sampled with the same temporal recurrence, so the first-observed year could be less precise in plot sampled with less frequency. However, when the species was first observed in the plot the first year the plot was sampled, it was not possible to determine the species colonisation year basing

on the previous sampled year. In this case, we followed one of the next two approaches to estimate colonisation year: 1) if the plot was abandoned shortly before the beginning of the surveys (i.e. less than 6 years before, which is the average gap between surveys), the colonisation year was estimated as the midpoint between year of species appearance in the plot and year of field abandonment; and 2) if the plot was abandoned more than 6 years before the beginning of the surveys, we estimated the colonisation year as the year of plot abandonment plus the species' colonisation delay. The colonisation delay is defined as the period of time that the species delay appearing after crop abandonment. We calculated it by estimating the difference between the species first-colonisation and the year of abandonment in those cases where it was possible to infer the colonisation year with high precision (i.e., the species appeared after the first survey, or the field was abandoned short before the appearance of the species in the plot). Then, used the mode (the most likely value) of this distribution of delay values as the species representative delay in colonisation. We used the mode instead the average or median since median or average values may not match a likely colonisation value in case of bi or multimodal distributions (Appendix S1-Fig. S1).

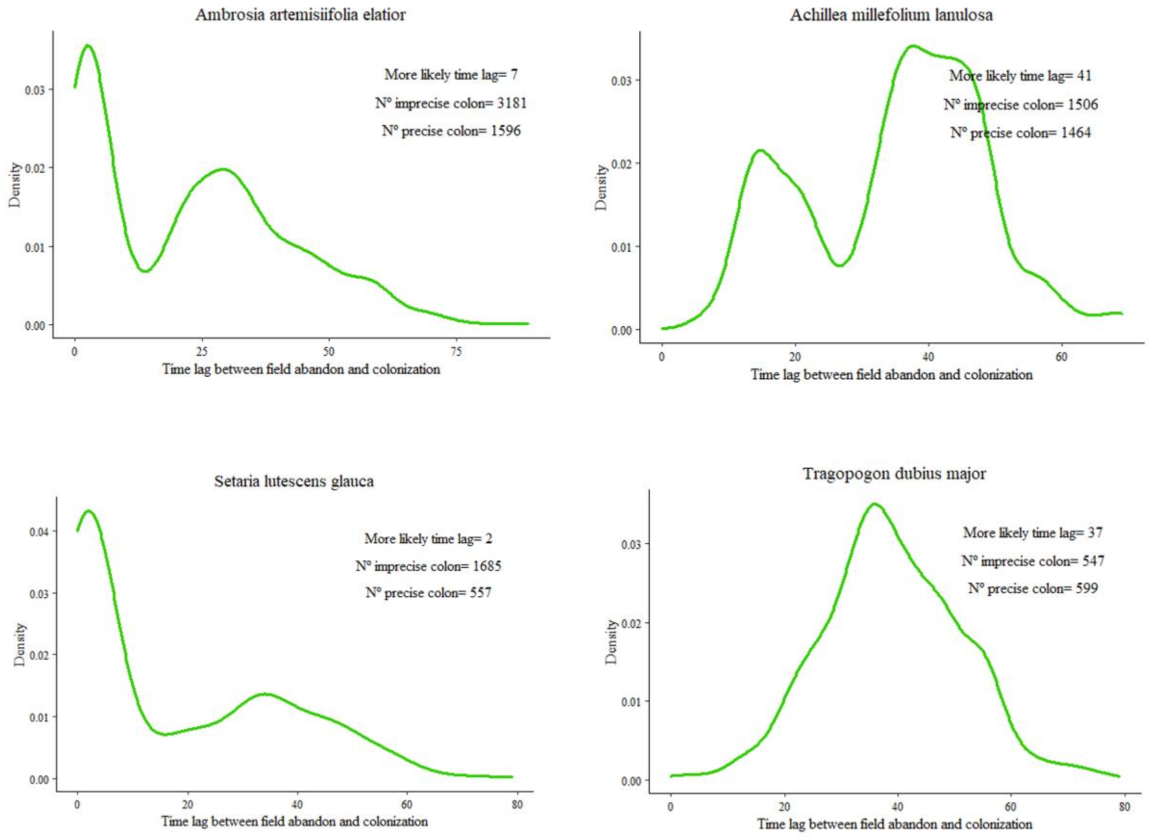

Section S1-Figure S1. Density plot of time lapse between cropland abandon and first documented occurrence in a given plot for different species. “Imprecise colon” referrer to the number of plots which appeared on the first sampling year and where the time lag between colonisation and abandon is higher than the usual lag between sampling years. “Precise colon” referrer to the number of plots which appeared after the first sampling year or appeared on the first sampled year but the time lag between colonisation and abandon is higher equal or lower the usual lag between sampling years. “More likely time lag” refers to the most probable number of years that a given species needs to colonize. Note that the ratio between the number of precise and imprecise colonisation cases suggests the robustness of imputation of time lag after abandon. The higher number of imprecise colonisation cases/number precise colonisation cases the less plausibility and robustness of the imputed value.

Section S2. Further details on trait data

Functional trait data for the study species were compiled from trait databases of five different studies carried out at Cedar Creek Ecosystem Science Reserve (Cadotte et al., 2009; Catford et al., 2019; Willis et al., 2010 and experiment e133). We compiled a total of 56 quantitative functional traits across species and datasets (Fig. S3 and S4). For each study dataset we averaged trait data at species level when more than one individual per species was sampled. Among the 56 total functional traits we selected seven that had the highest coverage across species: specific leaf area (SLA), plant height, leaf area, leaf dry matter content (LDMC), leaf fresh mass, leaf dry mass (all of them with data for more than 60% of the species) and seed mass (with data for 55% of the species). Although there were some missing values in the trait-species matrix, native and introduced species were equally well represented (Fig. S3). For taxa that could only be identified to genus level, we used average trait data across the species of the corresponding genus present in the study site. When a species had data for a given functional trait from more than one dataset, we choose the trait dataset following the next quality criteria: Catford et al. 2019>Cadotte et al. 2009>Willis et al. 2010> e133, attending to the number of replicates used to obtain the functional trait value (Catford between 3 and 15 replicates, Cadotte et al. 2009 10 replicates, Willis et al. 2010, and e133 3 replicates), the presence of interpolated data from other datasets (e133), and the publication status (published vs unpublished in peer-review journals). We did not average data across datasets to ensure the prevalence of maximum quality datasets. Finally, most of the trait data came from Catford et al. 2019 dataset.

Section S3. Appendix References

Cadotte, M.W., Cavender-Bares, J., Tilman, D. & Oakley, T.H. (2009). Using phylogenetic, functional and trait diversity to understand patterns of plant community productivity. *PLoS One*, 4, 1–9.

Catford, J.A., Smith, A.L., Wragg, P.D., Clark, A.T., Kosmala, M., Cavender-Bares, J., *et al.* (2019). Traits linked with species invasiveness and community invasibility vary with time, stage and indicator of invasion in a long-term grassland experiment. *Ecol. Lett.*, 22, 593–604.

Knops, J.M.H. & Bradley, K. (2009). Soil Carbon and Nitrogen Accumulation and Vertical Distribution

across a 74-Year Chronosequence. *Soil Sci. Soc. Am. J.*, 73, 2096–2104.

Knops, J.M.H. & Tilman, D. (2000). Dynamics of soil nitrogen and carbon accumulation for 61 years after agricultural abandonment. *Ecology*, 81, 88–98.

Willis, C.G., Halina, M., Lehman, C., Reich, P.B., Keen, A., McCarthy, S., *et al.* (2010). Phylogenetic community structure in Minnesota oak savanna is influenced by spatial extent and environmental variation. *Ecography (Cop.).*, 33, 565–577.

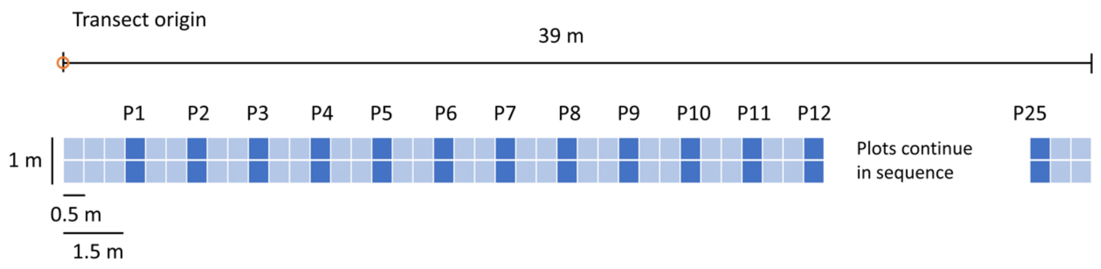

Figure S2. Transect spatial structure. P1 to P25 represent each of the plots nested within the transect. Dark blue represents the sampled plots and clear blue colour the non-sampled surface between plots. Figure adapted from Cedar Creek web page:

(https://cedarcreek.umn.edu/research/experiments/e014)

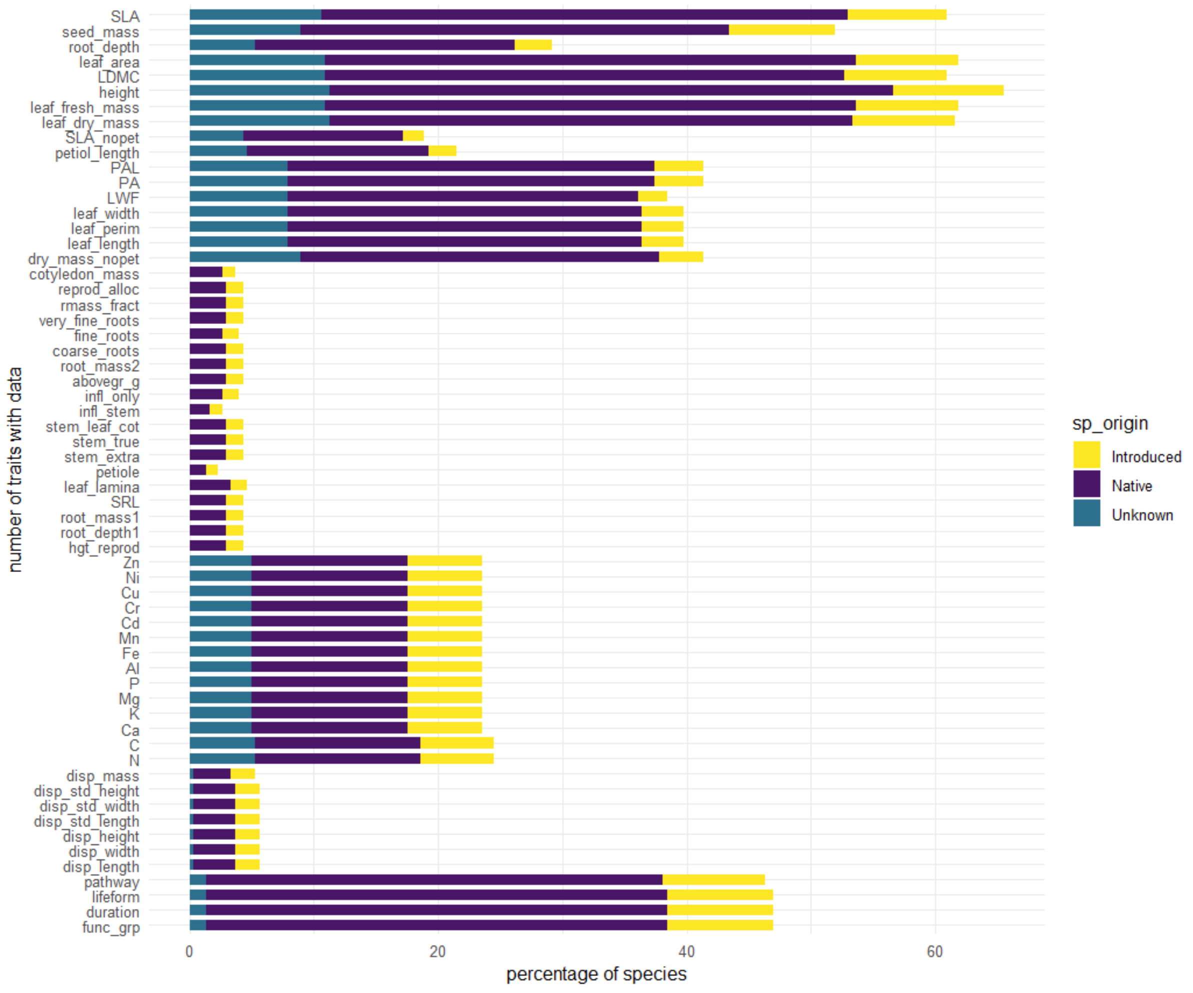

Figure S3. Percentage species with data for each trait variable per origin status. Different colours represent the species origin (yellow – introduced species, purple – native species and blue – species with unknown origin). Total number of species is 303.

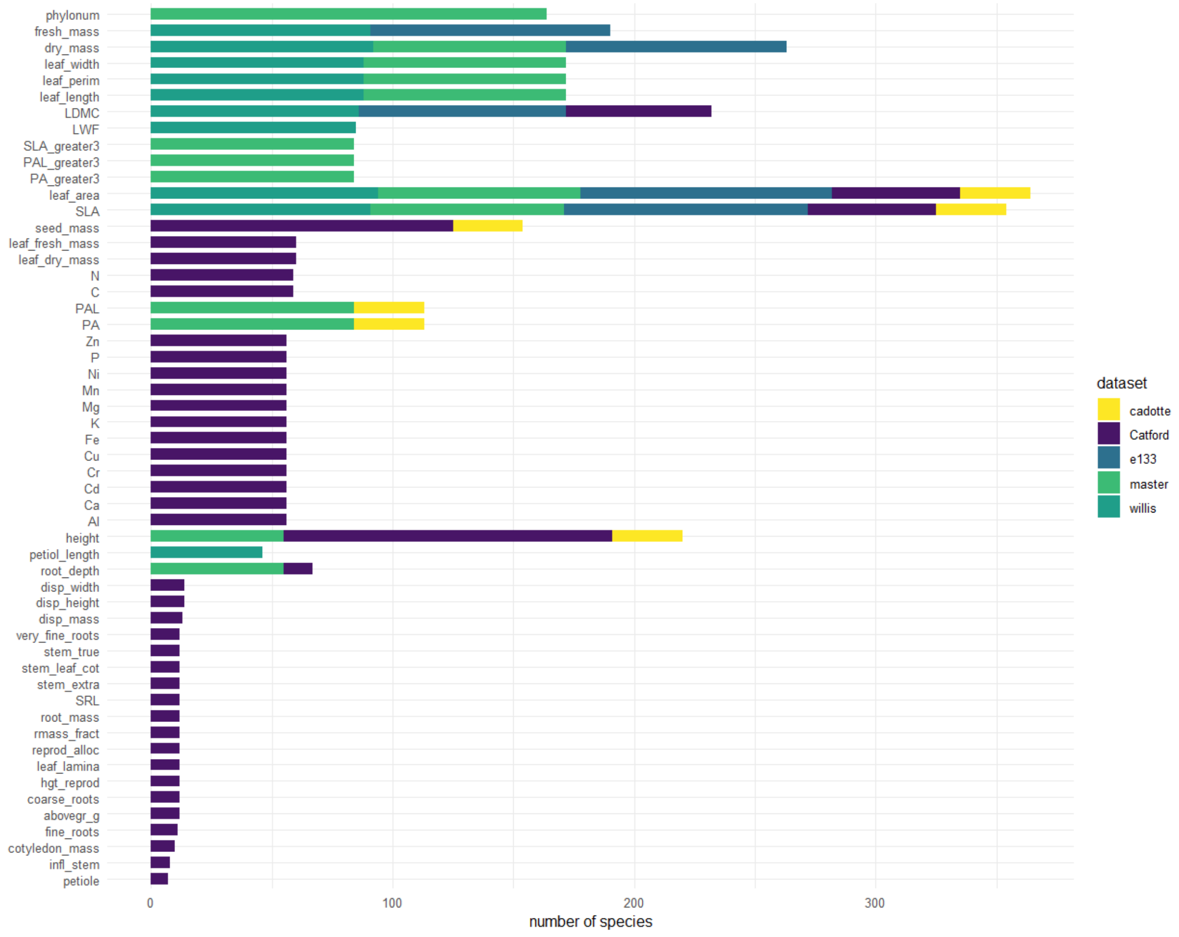

Figure S4. Number of species with trait data regarding to source trait dataset. In yellow Cadotte et al. 2009, in purple Catford et al. 2019, in dark blue e133 experiment data, in green unpublished data from a master thesis supervised by Willis, and in blue-green Willis et al. 2010. Please consider that a same species could have data from more than one dataset, so the sum of all the covered species by different datasets may be higher than the total species richness of the study (303 species).

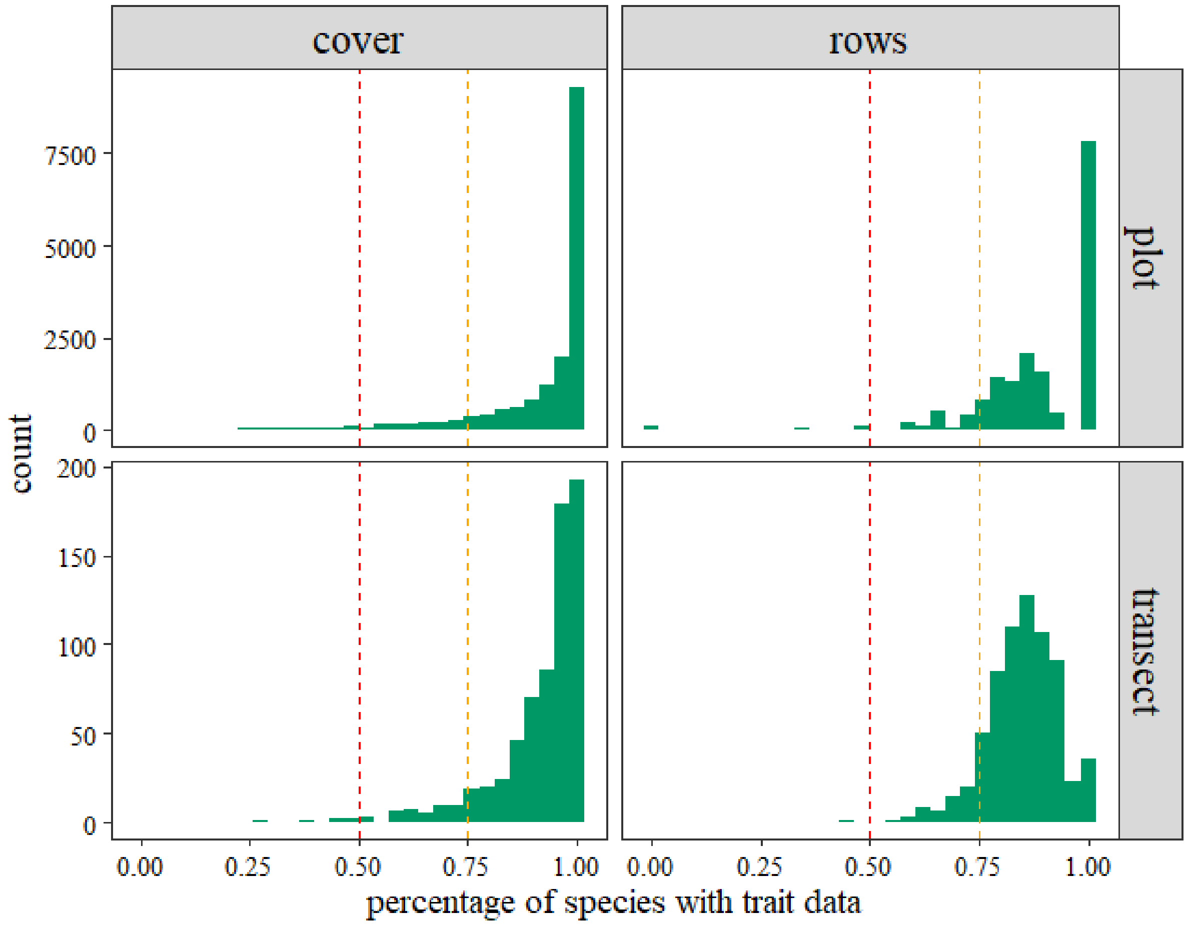

Figure S5. Proportion of species with functional trait data at plot and transect level from the total number of species present within each plot or transect. Left column shows frequencies regarding plant cover (i.e., Proportion of plot/transect cover with trait data) and right column shows frequencies regarding number of species (i.e., Table rows, at species and transect level). Red dashed line shows the threshold of 50% of species/cover having functional data per sampling unit, and orange dashed line shows the threshold of 75% of species/cover with functional data per sampling unit.

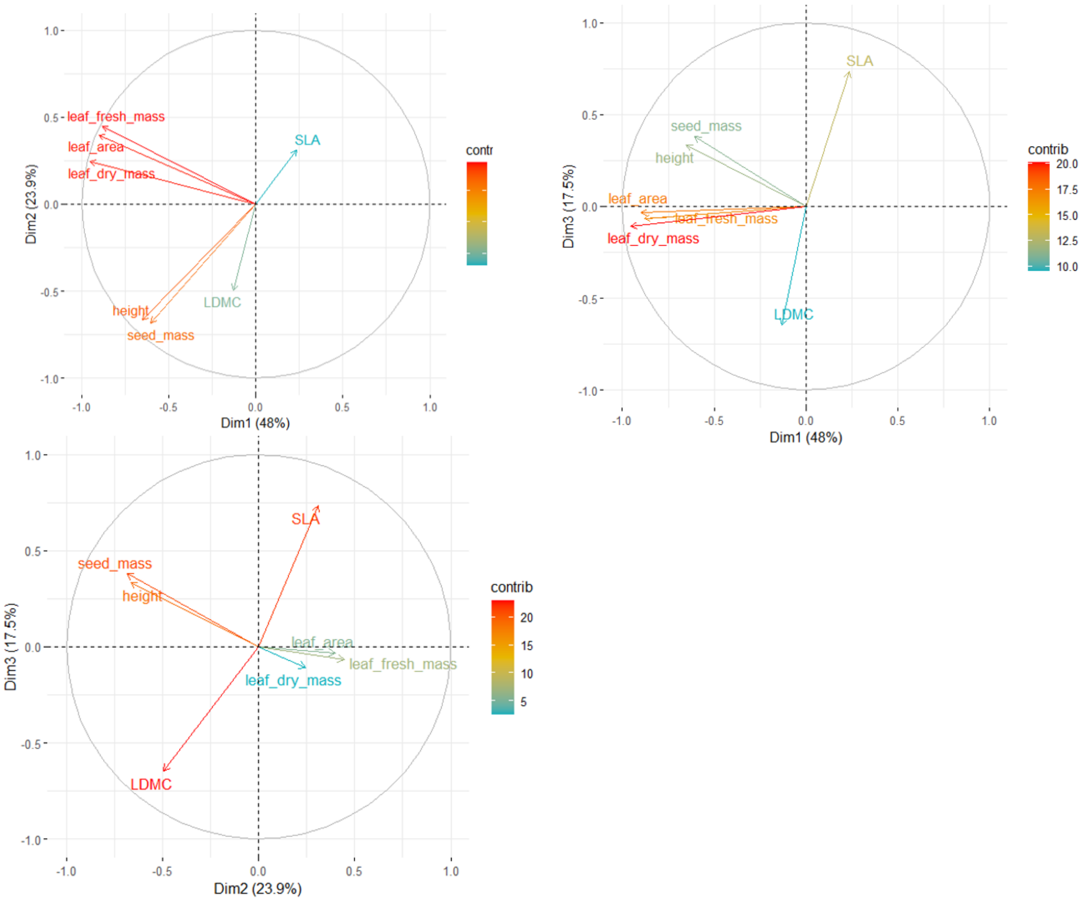

Figure S6. Correlation circles obtained from PCA for the 7 selected functional traits: SLA, seed mass, leaf area, LDMC, height, leaf fresh mass, and leaf dry mass. The three first axis accumulated the 75.37% of total variability. Top left panel represents the orthogonal distribution of functional traits in the bidimensional space of PCA dimensions 1 and 2; top right panel represents the orthogonal distribution of functional traits in the bidimensional space of PCA dimensions 1 and 3; and bottom left panel represents the orthogonal distribution of functional traits in the bidimensional space of PCA dimensions 2 and 3. Variables' colour represents the percentage of each variable contribution to the PCA.

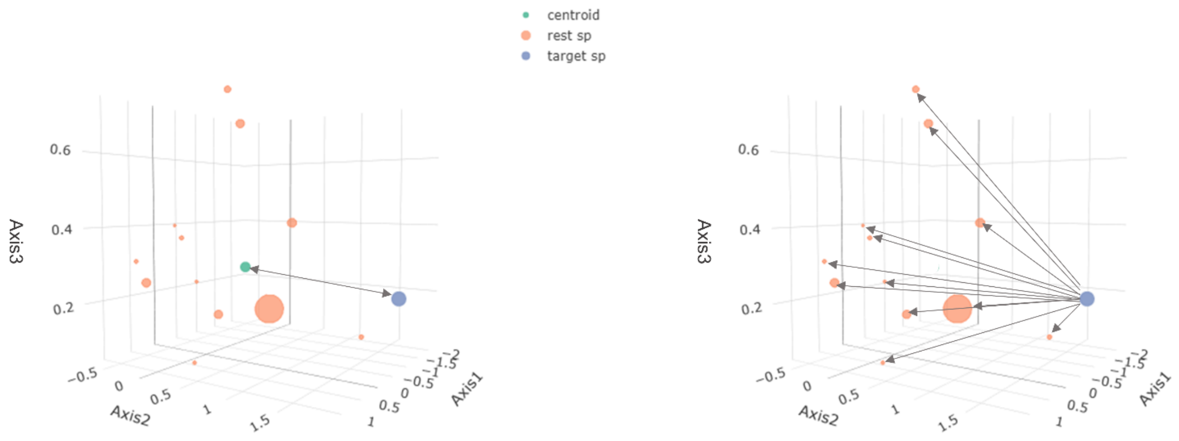

Figure S7. Diagram representing the approaches used to measure the functional dissimilarity in the three-dimensional (3D) functional multivariate trait space built with the seven selected functional variables: SLA, seed mass, leaf area, LDMC, height, leaf fresh mass, and leaf dry mass. Axis 1 explains 45.4% of the total traits' variance, while Axis 2 and Axis 3 explain 19.8% and 14.4% of the total variance, respectively. This diagram is an example built with species data from transect A, field 39 of 2016 sampled year. Blue point represents the target species position (Andropogon gerardii) in the functional multivariate space, while orange points represent the position of the rest of species of the same community. The green dot represents the 3D position of the community centroid of the transect community (estimated as the centre of mass of the rest of the species but the target species). Point size represents the species relative abundance within the community. In the left panel multivariate functional dissimilarity was estimated following the "centroid distance method", in which we measured the distance between the target species functional position in 3D and the position of the community centroid. In the right panel, multivariate functional dissimilarity was estimated following the "average pairwise distances", in which we measured the distance between the target species and each of the species present in the community, and then calculated the mean of these distances weighting by species relative cover.

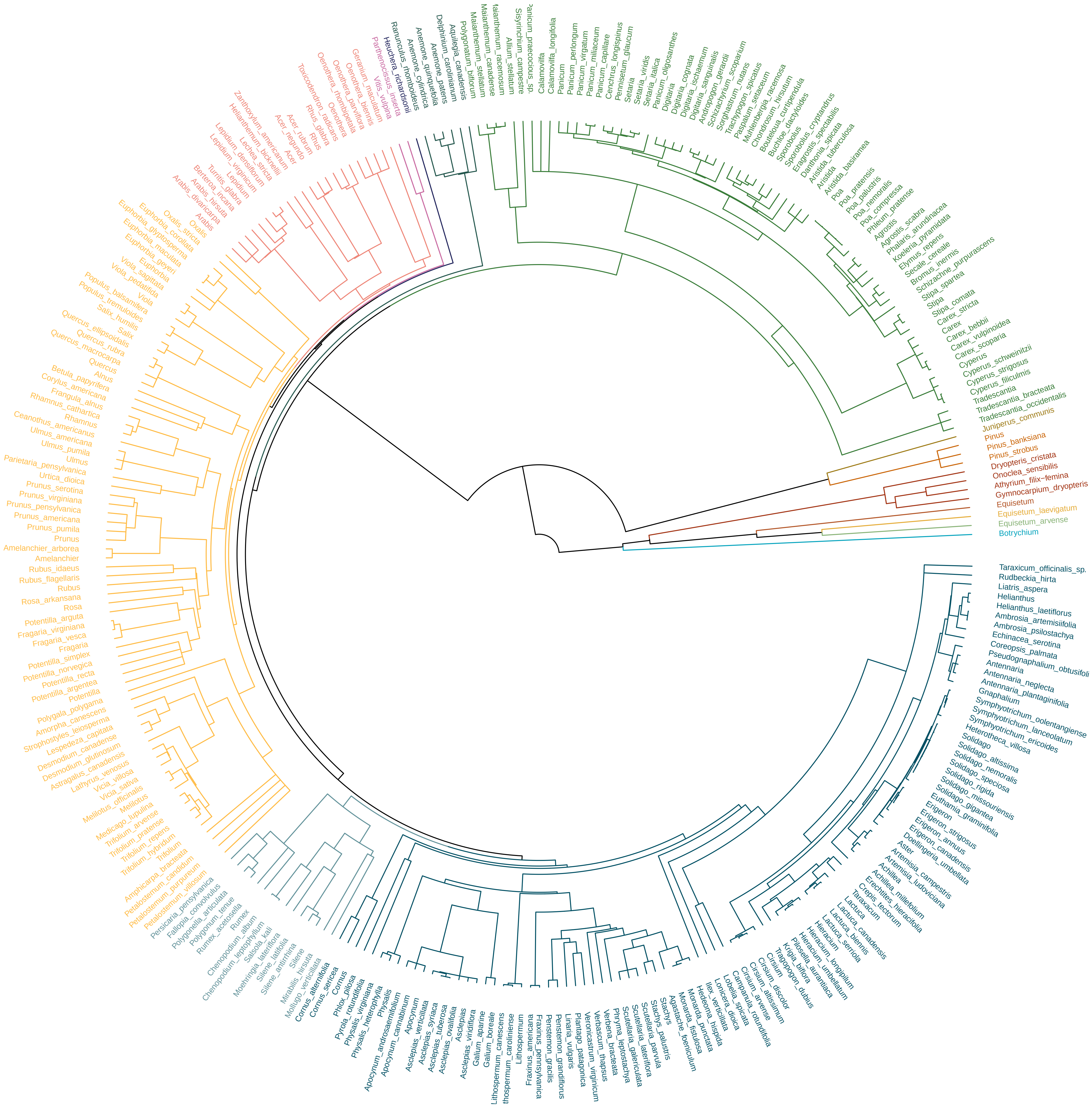

Taraxicum_officinalis_sp.
Rudbeckia_hirta
Liatris_aspera
Helianthus
Helianthus_laetiflorus
Ambrosia_artemisiifolia
Ambrosia_psilostachya
Echinacea_serotina
Coreopsis_palmata
Pseudognaphalium_obtusifoli
Antennaria
Antennaria_neglecta
Antennaria_plantaginifolia
Gnaphalium
Symphyotrichum_oolentangiense
Symphyotrichum_lanceolatum
Symphyotrichum_ericoides
Heterotheca_villosa
Solidago
Solidago_altissima
Solidago_nemoralis
Solidago_speciosa
Solidago_rigida
Solidago_missouriensis
Solidago_gigantea
Euthamia_graminifolia
Erigeron
Erigeron_strigosus
Erigeron_annuus
Erigeron_canadensis
Doellingeria_umbellata
Aster
Artemisia_campestris
Artemisia_ludoviciana
Achillea
Achillea_millefolium
Erechtites_hieraciifolia
Crepis_tectorum
Taraxacum
Lactuca
Lactuca_canadensis
Lactuca_biennis
Lactuca_serriola
Hieracium
Hieracium_longipilum
Hieracium_umbellatum
Pilosella_aurantiaca
Krigia_biflora
Tragopogon_dubius
Cirsium_discolor
Cirsium
Cirsium_altissimum
Cirsium_arvense
Campanula_rotundifolia
Lobelia_spicata
Lonicera_dioica
Ilex_verticillata
Hedeoma_hispida
Monarda_punctata
Monarda_fistulosa
Agastache_foeniculum
Stachys
Stachys_palustris
Scutellaria_parvula
Scutellaria_lateriflora
Scutellaria_galericulata
Phryma_leptostachya
Verbena_bracteata
Verbascum_thapsus
Veronicastrum_virginicum
Plantago_patagonica
Linaria_vulgaris
Penstemon_grandiflorus
Penstemon_gracilis
Fraxinus_pennsylvanica
Fraxinus_americana
Lithospermum
thospermum_caroliniense
Lithospermum_canescens
Galium_boreale
Galium_aparine
Asclepias_viridiflora
Asclepias
Asclepias_ovalifolia
Asclepias_tuberosa
Asclepias_syriaca
Asclepias_verticillata
Apocynum
Apocynum_cannabinum
Apocynum_androsaemifolium
Physalis
Physalis_heterophylla
Physalis_virginiana
Pyrola_rotundifolia
Phlox_pilosa
Cornus
Cornus_sericea
Cornus_alternifolia
Mollugo_verticillata
Mirabilis_hirsuta
Silene
Silene_antirrhina
Silene_latifolia
Moehringia_lateriflora
Salsola_kali
Chenopodium_leptophyllum
Chenopodium_album
Rumex
Rumex_acetosella
Polygonum_tenue
Polygonella_articulata
Fallopia_convolvulus
Persicaria_pensylvanica
Petalostemum_villosum
Petalostemum_purpureum
Petalostemum_candidum
Amphicarpa_bracteata
Trifolium
Trifolium_hybridum
Trifolium_repens
Trifolium_pratense
Trifolium_arvense
Medicago_lupulina
Melilotus
Melilotus_officinalis
Vicia_sativa
Vicia_villosa
Lathyrus_venosus
Astragalus_canadensis
Desmodium_glutinosum
Desmodium_canadense
Lespedeza_capitata
Strophostyles_leiosperma
Amorpha_canescens
Polygala_polygama
Potentilla
Potentilla_argentea
Potentilla_recta
Potentilla_norvegica
Potentilla_simplex
Fragaria
Fragaria_vesca
Fragaria_virginiana
Potentilla_arguta
Rosa
Rosa_arkansana
Rubus
Rubus_flagellaris
Rubus_idaeus
Amelanchier
Amelanchier_arborea
Prunus
Prunus_pumila
Prunus_americana
Prunus_pensylvanica
Prunus_virginiana
Prunus_serotina
Urtica_dioica
Parietaria_pensylvanica
Ulmus
Ulmus_pumila
Ulmus_americana
Ceanothus_americanus
Rhamnus
Rhamnus_cathartica
Frangula_alnus
Corylus_americana
Betula_papyrifera
Alnus
Quercus
Quercus_macrocarpa
Quercus_rubra
Quercus_ellipsoidalis
Salix
Salix_humilis
Populus_tremuloides
Populus_balsamifera
Viola
Viola_pedatifida
Viola_sagittata
Euphorbia
Euphorbia_geyeri
Euphorbia_maculata
Euphorbia_glyptosperma
Euphorbia_corollata
Oxalis_stricta
Oxalis
Arabis
Arabis_divaricarpa
Arabis_hirsuta
Berteroa_incana
Turritis_glabra
Lepidium
Lepidium_virginicum
Lepidium_densiflorum
Lechea_stricta
Helianthemum_bicknellii
Zanthoxylum_americanum
Acer
Acer_negundo
Acer_rubrum
Rhus
Rhus_glabra
Toxicodendron_radicans
Oenothera
Oenothera_rhombipetala
Oenothera_parviflora
Oenothera_biennis
Geranium_maculatum
Vitis_vulpina
Parthenocissus_inserta
Heuchera_richardsonii
Ranunculus_rhomboideus
Anemone_cylindrica
Anemone_quinquefolia
Anemone_patens
Delphinium_carolinianum
Aquilegia_canadensis
Polygonatum_biflorum
Maianthemum_stellatum
Maianthemum_canadense
faianthemum_racemosum
Allium_stellatum
Sisyrinchium_campestre
'anicum_praecocious_sp.
Calamovilfa
Calamovilfa_longifolia
Panicum
Panicum_perlongum
Panicum_virgatum
Panicum_miliaceum
Panicum_capillare
Cenchrus_longispinus
Pennisetum_glaucum
Setaria
Setaria_viridis
Setaria_italica
Panicum_oligosanthes
Digitaria
Digitaria_cognata
Digitaria_ischaemum
Digitaria_sanguinalis
Andropogon_gerardii
Schizachyrium_scoparium
Sorghastrum_nutans
Trachypogon_spicatus
Paspalum_setaceum
Muhlenbergia_racemosa
Chondrosum_hirsutum
Bouteloua_curtipendula
Buchloe_dactyloides
Sporobolus
Sporobolus_cryptandrus
Eragrostis_spectabilis
Danthonia_spicata
Aristida_tuberculosa
Aristida_basiramea
Aristida
Poa
Poa_pratensis
Poa_palustris
Poa_nemoralis
Poa_compressa
Phleum_pratense
Agrostis
Agrostis_scabra
Phalaris_arundinacea
Koeleria_pyramidata
Elymus_repens
Secale_cereale
Bromus_inermis
Schizachne_purpurascens
Stipa_spartea
Stipa
Stipa_comata
Carex_stricta
Carex
Carex_bebbii
Carex_vulpinoidea
Carex_scoparia
Cyperus
Cyperus_schweinitzii
Cyperus_strigosus
Cyperus_filiculmis
Tradescantia
Tradescantia_bracteata
Tradescantia_occidentalis
Juniperus_communis
Pinus
Pinus_banksiana
Pinus_strobus
Dryopteris_cristata
Onoclea_sensibilis
Athyrium_filix−femina
Gymnocarpium_dryopteris
Equisetum
Equisetum_laevigatum
Equisetum_arvense
Botrychium

Figure S8. Phylogenetic tree for all the 303 species included in e014 Cedar Creek experiment database based on Smith and Brown 2018 and Zanne et al. (2014) phylogeny.

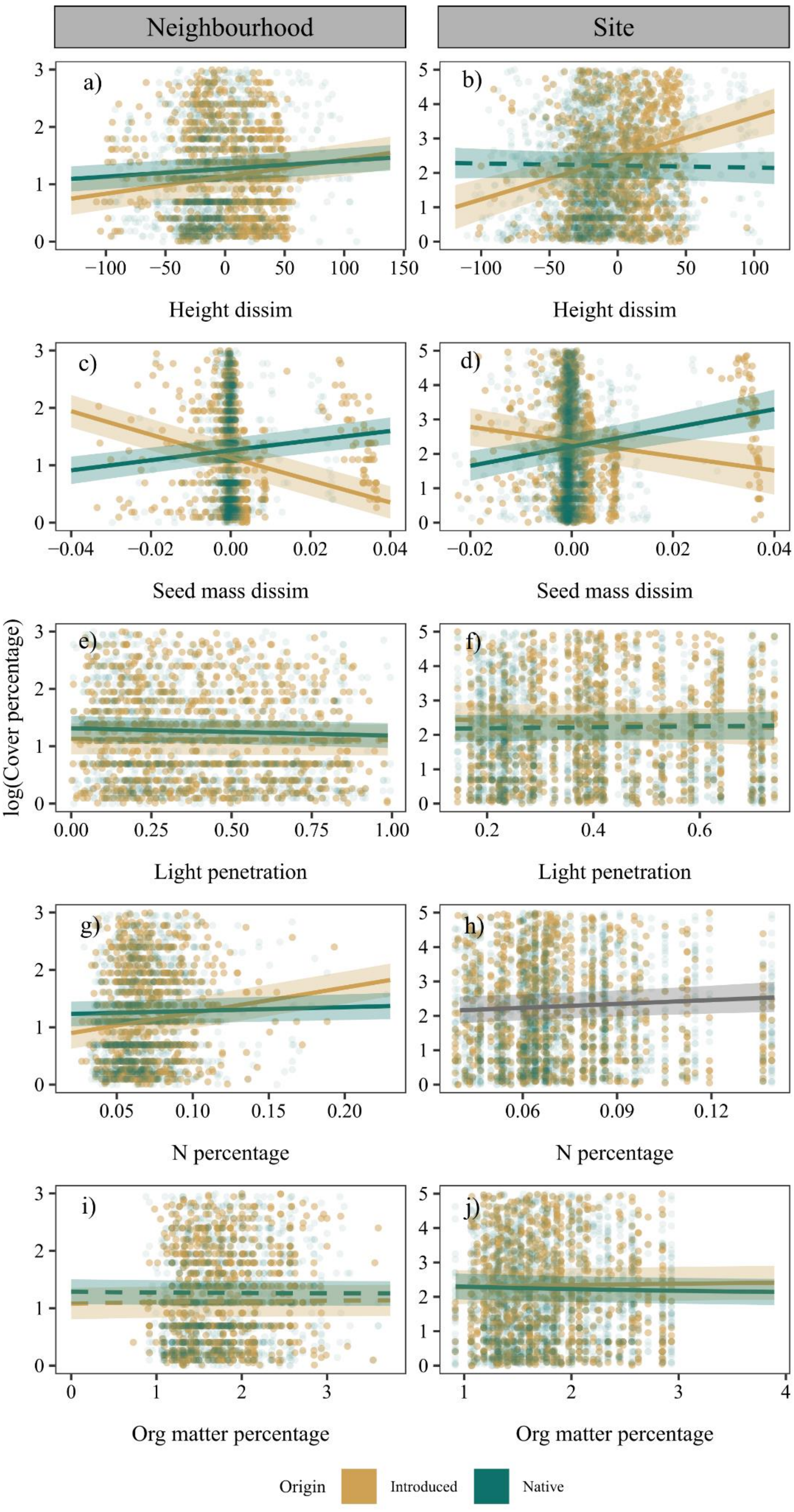
Neighbourhood
Site
a)
b)
c)
d)
e)
f)
g)
h)
i)
j)
log(Cover percentage)
Height dissim
Seed mass dissim
Light penetration
N percentage
Org matter percentage
Origin
Introduced
Native

Figure S9. Regression plots corresponding to final selected model for both neighbourhood and site spatial scales estimating dissimilarity as ranked trait differences. Y axes represent log of species cover percent. X axes correspond to each of the explanatory variables. Bluegreen color represents native species populations and brown color represents native species populations. Grey color represents common trends for all the species when there were no significant differences between native and introduced species. Solid and dashed lines represent significant ($p < 0.05$) and not significant effects ($p > 0.05$), respectively. Plots only includes 5% of species x plot raw data used in models at neighbourhood level and 25% of species x transect raw data at site level, in order to reduce the number of points (to 5000 each) and improve visualization.

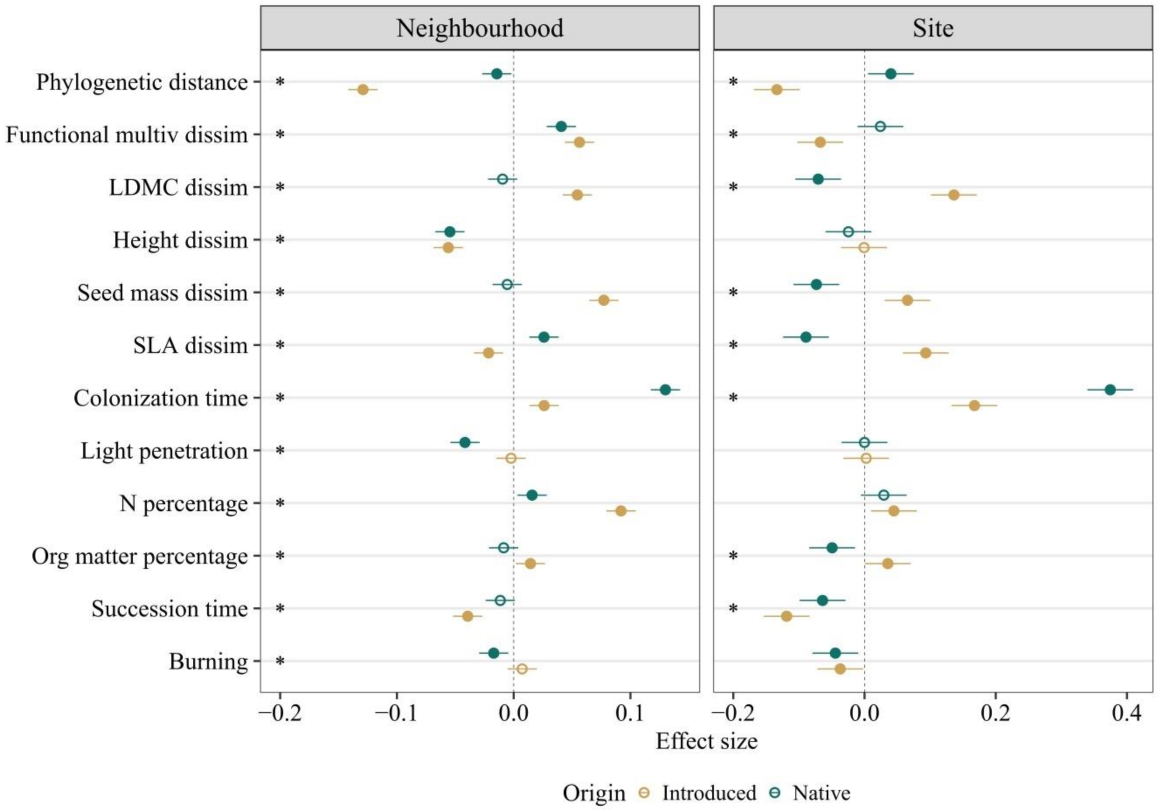

Figure S10. Effect size with 95% confidence intervals of each single explanatory variable included in the final selected model both for neighbourhood (0.5 m2) and site spatial scales (~40 m2) considering dissimilarity as pairwise absolute differences. See Table S2 for further details on model structure and variable transformation. Dark green dots represent native species, and ochre dots represent introduced species. Filled dots and empty dots represent statistically significant ($p < 0.05$) and non-significant effects ($p > 0.05$), respectively. Asterisk in the left of each panel indicates the presence of statistically significant differences between effect sizes of native and introduced species.

Table S1. Definitions and units of measurement of traits included in the study.

Trait	Unit	Comments
**fresh_mass**	g	Fresh lamina mass including petiole (g)
**dry_mass**	g	Dry lamina mass including petiole (g)
**LDMC**	mg/g	Lamina dry matter content including petiole (mg/g)
**Leaf area**	mm^2	fresh leaf area (scanned)
**SLA**	mm^2/mg	area/(dry_mass*1000)
**plant height**	cm	vegetative height
**seed mass**	g	seed mass for a seed sample/number of seeds

Table S2 Results of Blomberg's K test to assess the phylogenetic signal of the seven functional traits used to build the multivariate functional space. The table also includes the Pearson's correlation between multivariate functional dissimilarity (FMD) and phylogenetic distance (PD).

Trait	Blomberg's K	Correlation FMD-PD Neighbourhood scale	Correlation FMD-PD Site scale
**Height**	0.093	0.150	0.117
**SLA**	0.039		
**LDMC**	0.112		
**Seed mass**	0.339		
**Leaf fresh mass**	0.022		
**Leaf dry mass**	0.014		
**Leaf area**	0.022		

Table S3. Variables included in each of the 6 tested alternative models. Variables were selected after discarding regression multicollinearity. Thus, carbon percentage, dissimilarity in leaf area, dissimilarity in dry matter content and dissimilarity in leaf fresh mass are not included in the table as they were discarded from models including environmental variables and univariate functional dissimilarity, respectively, due to high collinearity. Green colour shading represents environmental variables, orange colour shading represent functional trait variables, yellow colour represents phylogenetic variables and blue colour represent temporal variables. Final selected model corresponds to model 4.

Variable code	Variable	Transformation	Model 1	Model 2	Model 3	Model 4	Model 5
PD	Phylogenetic distance	scaled	x	x	x	x	x
MFD	Multivariate functional dissimilarity	log	x	x	x	x	x
UFD_sm	Seed mass dissimilarity	log			x	x	x
UFD_sla	specific leaf area dissimilarity	scaled			x	x	x
UDF_ldmc	leaf dry matter content dissimilarity	scaled			x	x	x
UFD_h	plant height dissimilarity	scaled			x	x	x
ENV_n	Soil nitrogen content			x		x	x
ENV_c	Soil carbon content						x
ENV_om	Soil organic matter content	log		x		x	x
ENV_b	Burning treatment			x		x	x
ENV_lp	Light penetration			x		x	x
TEMP_suc	Time since abandonment	scaled		x		x	
TEMP_colon	Minimum Colonisation Time	log		x		x	x
ENV_tab_int	Time since abandonment Interacting with each of the rest explanatory variables	scaled					x

Table S4. Model summary for each of the fitted models at neighbourhood level (plot scale) using ranked functional univariate dissimilarities. Table shows: R^2 marginal (**R^2 m**), R^2 conditional (**R^2 c**), Akaike Information Criterion (AIC) and the difference between each model AIC, the difference between model AIC and the minimum AIC value across models (AIC dif), Bayesian Information Criterion (BIC) and BIC difference between each model BIC and the lowest BIC found across models. Final selected model and further results corresponds to model 4.

MODEL	R^2M	R^2C	AICc	AICc_DIF	BIC	BIC_DIF
**MODEL 1**	0.005	0.378	273691.58	25915.44	273806.44	25659.51
**MODEL 2**	0.027	0.313	248893.67	1117.54	249121.48	974.55
**MODEL 3**	0.046	0.413	272735.90	24901.50	272927.40	24682.90
**MODEL 4**	**0.077**	**0.358**	247902.73	67.6	**248206.47**	**0**
**MODEL 5**	0.050	0.320	247834.4	0	248244.5	38.03

Table S5. Model summary for each of the fitted models at site level (transect scale) using ranked functional univariate dissimilarities. Table shows: R^2 marginal (**R^2 m**), R^2 conditional (**R^2 c**), Akaike Information Criterion (AIC) and the difference between each model AIC, the difference between model AIC and the minimum AIC value across models (AIC dif), Bayesian Information Criterion (BIC) and BIC difference between each model BIC and the lowest BIC found across models. Final selected model and further results corresponds to model 4.

MODEL	R^2M	R^2C	AICC	AICC_DIF	BIC	BIC_DIF
**MODEL 1**	0.015	0.395	46634.44	4432.53	46717.11	4284.61
**MODEL 2**	0.065	0.431	42535.68	333.77	42706.88	274.38
**MODEL 3**	0.134	0.487	46223.05	3892.27	46373.26	3737.158
**MODEL 4**	**0.175**	**0.495**	**42203.74**	**0.00**	**42434.32**	**0.00**
**MODEL 5**	0.101	0.419	42330.78	127.04	42635.68	201.36

Table S6. Model summary for each of the fitted models at plot level (neighbourhood scale) using absolute pairwise functional univariate dissimilarities. Table shows: R^2 marginal (**R^2 m**), R^2 conditional (**R^2 c**), Akaike Information Criterion (AIC) and the difference between each model AIC, the difference between model AIC and the minimum AIC value across models (AIC dif), Bayesian Information Criterion (BIC) and BIC difference between each model BIC and the lowest BIC found across models. Final selected model and further results corresponds to model 4.

MODEL	R^2M	R^2C	AICC	AICC_DIF	BIC	BIC_DIF
**MODEL 1**	0.009	0.387	273473.1	25032.7	273588.0	24843.9
**MODEL 2**	0.030	0.318	248818.8	378.4	249046.6	302.5
**MODEL 3**	0.016	0.395	273062.2	24621.8	273253.7	24509.6
**MODEL 4**	**0.037**	**0.325**	**248440.4**	**0**	**248744.1**	**0**
**MODEL 5**	0.036	0.322	248597.1	156.7	248995.8	251.7

Table S7. Model summary for each of the fitted models at site level (transect scale) using absolute pairwise functional univariate dissimilarities. Table shows: R^2 marginal (**R^2 m**), R^2 conditional (**R^2 c**), Akaike Information Criterion (AIC) and the difference between each model AIC, the difference between model AIC and the minimum AIC value across models (AIC dif), Bayesian Information Criterion (BIC) and BIC difference between each model BIC and the lowest BIC found across models. Final selected model and further results corresponds to model 4.

MODEL	R^2M	R^2C	AICC	AICC_DIF	BIC	BIC_DIF
**MODEL 1**	0.010	0.403	46498.78	4213.15	46581.4	24843.9
**MODEL 2**	0.060	0.435	42398.88	113.25	42569.98	302.5
**MODEL 3**	0.064	0.455	46262.47	3976.84	46412.67	24509.6
**MODEL 4**	**0.080**	**0.457**	**42285.63**	**0**	**42516.22**	**0**
**MODEL 5**	0.069	0.430	42389.97	104.34	42694.87	251.7

Table S8. Model results for the finally selected model at neighbourhood spatial scale including ranked functional dissimilarities. The table presents variable slopes separately for native and introduced species when their effects differ significantly. If the species origin interaction is not significant, a common slope for all species is reported. Bold pvalue highlights variables' slopes significantly different from 0 slope (pv<0.05).

Variable	Origin	Slope	SE	z.ratio	p.value
**Succession time**	Introduced	-0.010	0.002	-5.751	**<0.001**
**Succession time**	Native	-0.003	0.002	-1.695	0.090
**Minimum colonisation time**	Introduced	0.001	0.000	3.640	**<0.001**
**Minimum colonisation time**	Native	0.006	0.000	20.446	**<0.001**
**Phylogenetic distance**	Introduced	-0.002	0.000	-24.158	**<0.001**
**Phylogenetic distance**	Native	0.000	0.000	-1.456	0.145
**Functional multivariate dissimilarity**	Intro/Native	0.019	0.003	6.097	**<0.001**
**N percentage in soil**	Introduced	4.406	0.287	15.358	**<0.001**
**N percentage in soil**	Native	0.657	0.269	2.442	**0.015**
**Org matter percentage in soil**	Introduced	0.012	0.006	1.922	0.055
**Org matter percentage in soil**	Native	-0.006	0.006	-0.932	0.352
**Light penetration**	Introduced	-0.020	0.023	-0.875	0.381
**Light penetration**	Native	-0.129	0.021	-6.195	**<0.001**
**SLA ranked dissimilarity**	Introduced	-0.006	0.001	-7.267	**<0.001**
**SLA ranked dissimilarity**	Native	-0.015	0.001	-15.795	**<0.001**
**LDMC ranked dissimilarity**	Introduced	0.001	0.000	6.411	**<0.001**
**LDMC ranked dissimilarity**	Native	-0.001	0.000	-6.390	**<0.001**
**Height ranked dissimilarity**	Introduced	0.003	0.000	9.464	**<0.001**
**Height ranked dissimilarity**	Native	0.001	0.000	6.367	**<0.001**
**Seed mass ranked dissimilarity**	Introduced	-19.853	1.059	-18.750	**<0.001**
**Seed mass ranked dissimilarity**	Native	8.558	1.345	6.362	**<0.001**
**Burning treatment**	Introduced	0.035	0.023	1.551	0.121
**Burning treatment**	Native	-0.063	0.022	-2.793	**0.005**

Table S9. Model results for the finally selected model at site spatial scale including ranked functional dissimilarities. The table presents variable slopes separately for native and introduced species when their effects differ significantly. If the species origin interaction is not significant, a common slope for all species is reported Bold pvalue highlights variables' slopes significantly different from 0 slope (pv<0.05).

Variable	Origin	Slope	SE	z.ratio	p.value
**Succession time**	Introduced	-0.015	0.003	-5.311	**<0.001**
**Succession time**	Native	-0.006	0.003	-2.336	**0.019**
**Minimum colonization time**	Introduced	0.013	0.001	9.944	**<0.001**
**Minimum colonization time**	Native	0.018	0.001	21.335	**<0.001**
**Phylogenetic distance**	Introduced	-0.006	0.001	-9.297	**<0.001**
**Phylogenetic distance**	Native	0.001	0.000	2.373	**0.018**
**Functional multivariate dissimilarity**	Intro/Native	-0.065	0.023	-2.781	**0.005**
**N percentage in soil**	Intro/Native	3.904	1.536	2.542	**0.011**
**Org matter percentage in soil**	Introduced	0.045	0.021	2.170	**0.030**
**Org matter percentage in soil**	Native	-0.054	0.019	-2.844	**0.004**
**Light penetration**	Introduced	-0.396	0.265	-1.495	0.135
**Light penetration**	Native	0.136	0.231	0.590	0.555
**SLA ranked dissimilarity**	Introduced	-0.043	0.005	-8.108	**<0.001**
**SLA ranked dissimilarity**	Native	-0.029	0.005	-6.284	**<0.001**
**LDMC ranked dissimilarity**	Intro/Native	0.001	0.001	1.680	0.093
**Height ranked dissimilarity**	Introduced	0.012	0.002	6.355	**<0.001**
**Height ranked dissimilarity**	Native	-0.001	0.001	-0.519	0.604
**Seed mass ranked dissimilarity**	Introduced	-21.342	6.403	-3.333	**0.001**
**Seed mass ranked dissimilarity**	Native	27.691	5.622	4.926	**<0.001**
**Burning treatment**	Intro/Native	0.087	0.030	0.562	**0.005**

Table S10. Model results for the finally selected model at neighbourhood spatial scale including absolute pairwise functional dissimilarities. The table presents variable slopes separately for native and introduced species when their effects differ significantly. If the species origin interaction is not significant, a common slope for all species is reported Bold pvalue highlights variables' slopes significantly different from 0 slope (pv<0.05).

Variable	Origin	Slope	SE	z.ratio	p.value
**Succession time**	Introduced	-0.011	0.002	-6.160	**<0.001**
**Succession time**	Native	-0.003	0.002	-1.797	0.072
**Minimum colonization time**	Introduced	0.001	0.000	4.078	**<0.001**
**Minimum colonization time**	Native	0.006	0.000	20.339	**<0.001**
**Phylogenetic distance**	Introduced	-0.002	0.000	-20.197	**<0.001**
**Phylogenetic distance**	Native	0.000	0.000	-2.262	**0.024**
**Functional multivariate dissimilarity**	Introduced	0.077	0.009	8.815	**<0.001**
**Functional multivariate dissimilarity**	Native	0.039	0.006	6.382	**<0.001**
**N percentage in soil**	Introduced	4.147	0.288	14.386	**<0.001**
**N percentage in soil**	Native	0.666	0.270	2.469	**0.014**
**Org matter percentage in soil**	Introduced	0.014	0.006	2.249	**0.025**
**Org matter percentage in soil**	Native	-0.009	0.006	-1.345	0.178
**Light penetration**	Introduced	-0.008	0.023	-0.354	0.724
**Light penetration**	Native	-0.136	0.021	-6.524	**<0.001**
**SLA pairwise dissimilarity**	Introduced	-0.004	0.001	-3.366	**0.001**
**SLA pairwise dissimilarity**	Native	0.005	0.001	4.058	**<0.001**
**LDMC pairwise dissimilarity**	Introduced	0.001	0.000	8.537	**<0.001**
**LDMC pairwise dissimilarity**	Native	0.000	0.000	-1.485	0.137
**Height pairwise dissimilarity**	Introduced	-0.003	0.000	-8.766	**<0.001**
**Height pairwise dissimilarity**	Native	-0.002	0.000	-8.549	**<0.001**
**Seed mass pairwise dissimilarity**	Introduced	11.939	0.988	12.084	**<0.001**
**Seed mass pairwise dissimilarity**	Native	-1.123	1.300	-0.864	0.387
**Burning treatment**	Introduced	0.026	0.023	1.133	0.257
**Burning treatment**	Native	-0.062	0.023	-2.677	**0.007**

Table S11. Model results for the finally selected model at site spatial scale including absolute pairwise functional dissimilarities. The table presents variable slopes separately for native and introduced species when their effects differ significantly. If the species origin interaction is not significant, a common slope for all species is reported Bold pvalue highlights variables' slopes significantly different from 0 slope (pv<0.05).

Variable	Origin	Slope	SE	z.ratio	p.value
**Succesion time**	Introduced	-0.022	0.003	-6.671	**<0.001**
**Succesion time**	Native	-0.011	0.003	-3.595	**<0.001**
**Minimum colonization time**	Introduced	0.012	0.001	9.416	**<0.001**
**Minimum colonization time**	Native	0.018	0.001	21.047	**<0.001**
**Phylogenetic distance**	Introduced	-0.005	0.001	-7.505	**<0.001**
**Phylogenetic distance**	Native	0.001	0.000	2.254	**0.024**
**Functional multivariate dissimilarity**	Introduced	-0.218	0.057	-3.789	**<0.001**
**Functional multivariate dissimilarity**	Native	0.043	0.031	1.361	0.174
**N percentage in soil**	Intro/Native	4.018	1.772	2.266	**0.023**
**Org matter percentage in soil**	Introduced	0.046	0.023	1.997	**0.046**
**Org matter percentage in soil**	Native	-0.059	0.021	-2.775	**0.006**
**Light penetration**	Intro/Native	0.014	0.249	0.057	0.954
**SLA pairwise dissimilarity**	Introduced	0.044	0.008	5.241	**<0.001**
**SLA pairwise dissimilarity**	Native	-0.033	0.007	-5.014	**<0.001**
**LDMC pairwise dissimilarity**	Introduced	0.007	0.001	7.653	**<0.001**
**LDMC pairwise dissimilarity**	Native	-0.002	0.001	-3.968	**<0.001**
**Height pairwise dissimilarity**	Intro/Native	-0.026	0.023	-1.121	0.262
**Seed mass pairwise dissimilarity**	Introduced	23.289	6.330	3.679	**<0.001**
**Seed mass pairwise dissimilarity**	Native	-24.357	5.913	-4.119	**<0.001**
**Burning treatment**	Intro/Native	-0.097	0.034	-1.551	**0.006**

Table S12. Differences in slope between introduced and native species slopes for each variable (introduced slope-native slope) estimating functional dissimilarities as ranked traits dissimilarities. Negative values mean higher native slopes while positive values imply higher introduced slopes. Bold pvalue highlights significant differences (pv<0.05).

	Neighbourhood scale			Site scale		
**Variable**	**Slope difference**	**z.ratio**	**p.value**	**Slope difference**	**z.ratio**	**p.value**
**Succession time**	-0.007	-15.800	**<0.001**	-0.009	-4.480	**<0.001**
**Minimum colonization time**	-0.005	-10.800	**<0.001**	-0.005	-3.330	**0.001**
**Phylogenetic distance**	-0.002	-16.400	**<0.001**	-0.007	-8.940	**<0.001**
**Functional multivariate dissimilarity**	0.006	0.898	0.369	0.024	0.439	0.661
**N percentage in soil**	3.750	11.500	**<0.001**	0.357	0.177	0.860
**Org matter percentage in soil**	0.018	2.990	**0.003**	0.099	4.190	**<0.001**
**Light penetration**	0.109	3.770	**<0.001**	-0.532	-2.150	**0.031**
**SLA ranked dissimilarity**	0.009	7.050	**<0.001**	-0.015	-2.330	**0.020**
**LDMC ranked dissimilarity**	0.002	9.400	**<0.001**	0.001	1.060	0.288
**Height ranked dissimilarity**	0.002	4.430	**<0.001**	0.013	6.170	**<0.001**
**Seed mass ranked dissimilarity**	-28.400	-17.300	**<0.001**	-49.000	-6.270	**<0.001**
**Burning treatment**	-0.193	-1.780	0.075	0.107	0.510	0.957

Table S13. Differences in slope between introduced and native species slopes for each variable (introduced slope-native slope) estimating functional dissimilarities as absolute pairwise dissimilarities. Negative values mean higher native slopes while positive values imply higher introduced slopes. Bold pvalue highlights significant differences (pv<0.05).

	neighbourhood scale			site scale		
**variable**	**slope difference**	**z.ratio**	**p.value**	**slope difference**	**z.ratio**	**p.value**
**Succession time**	-0.008	-17.600	**<0.001**	-0.011	-5.400	**<0.001**
**Minimum colonization time**	-0.004	-10.400	**<0.001**	-0.006	-3.610	**<0.001**
**Phylogenetic distance**	-0.002	-13.700	**<0.001**	-0.006	-7.500	**<0.001**
**Functional multivariate dissimilarity**	0.038	3.630	**<0.001**	-0.261	-4.030	**<0.001**
**N percentage in soil**	3.480	10.700	**<0.001**	2.330	1.170	0.241
**Org matter percentage in soil**	0.023	3.790	**<0.001**	0.105	4.390	**<0.001**
**Light penetration**	0.128	4.420	**<0.001**	0.044	0.179	0.858
**SLA pairwise dissimilarity**	-0.010	-5.330	**<0.001**	0.077	7.490	**<0.001**
**LDMC pairwise dissimilarity**	0.001	7.400	**<0.001**	0.009	8.620	**<0.001**
**Height pairwise dissimilarity**	-0.001	-2.000	**0.046**	0.002	0.765	0.444
**Seed mass pairwise dissimilarity**	13.100	8.370	**<0.001**	47.600	6.040	**<0.001**
**Burning treatment**	-0.318	-2.940	**0.017**	-0.325	-1.540	0.415
